# Supplementary material for: Orbitrap noise structure and method for noise unbiased multivariate analysis
Source: Nat Commun. 2025 Jul 10;16:6398. doi: 10.1038/s41467-025-61542-2 (PMC12246260; doi:10.1038/s41467-025-61542-2)
Supplement: Supplementary file 1 — Supplementary Information [file 41467_2025_61542_MOESM1_ESM.pdf]

Supplementary Information

Orbitrap noise structure and method for noise unbiased multivariate analysis

Michael R. Keenan[1†], Gustavo F. Trindade[2†], Alexander Pirkl[3], Clare L. Newell[4], Yuhong Jin[4], Konstantin Aizikov[5], Andreas Dannhorn[6], Junting Zhang[2], Lidija Matjačić[2], Henrik Arlinghaus[3], Anya Eyres[2], Rasmus Havelund[2], Richard J.A. Goodwin[6], Zoltan Takats[7], Josephine Bunch[2], Alex P. Gould[4], Alexander Makarov[5,8] and Ian S. Gilmore[2*]

† *These authors had equal contribution*

* *Corresponding author: ian.gilmore@npl.co.uk*

[1] Independent, Georgetown, Texas, USA
[2] National Physical Laboratory, NiCE-MSI, Teddington, UK
[3] IONTOF GmbH, Münster, Germany
[4] The Francis Crick Institute, London, UK
[5] Thermo Fisher Scientific, Bremen, Germany
[6] AstraZeneca, Cambridge, UK
[7] Imperial College London, London, UK
[8] Biomolecular Mass Spectrometry and Proteomics, Bijvoet Center for Biomolecular Research and Utrecht Institute for Pharmaceutical Sciences, University of Utrecht, Utrecht, The Netherlands

Contents

Supplementary Note 1: Expanded description of the data distribution for OrbiSIMS mass spectrometry 1
Supplementary Note 2: Experimental details for silver measurements 18
Supplementary Note 3: Further description of measured silver signal and unmodeled signal variations 19
Supplementary Note 4: Spectral profiles, centroids and peak lists 26
Supplementary Note 5: Statistics of binned noise 31
Supplementary Note 6: Simulation of the silver profiles and thresholding 34
Supplementary Note 7: Description of data scaling and transformations for multivariate analysis and comparison using silver datasets 39
Supplementary Note 8: Experimental details of biological imaging datasets 53
Supplementary Note 9: Additional figures 54

Supplementary Note 1: Expanded description of the data distribution for OrbiSIMS mass spectrometry

Statistical properties of Orbitrap™ MS signals and noise

The statistical distribution of data from an FT-based acquisition has been considered in the context of MRI imaging[1] and it accommodates the full range of S/N. For a constant signal magnitude S and time-domain noise standard deviation σ, the data are found to follow the Rician distribution[2] having the density and distribution functions:

$$X \sim Rician(S, \sigma)$$

$$f_X(x) = \frac{x}{\sigma^2} exp\left(-\frac{x^2 + S^2}{2\sigma^2}\right) I_0\left(\frac{Sx}{\sigma^2}\right) H(x) = g_X(x) H(x) \tag{1}$$

$$F_X(x) = 1 - Q_1\left(\frac{S}{\sigma}, \frac{x}{\sigma}\right)$$

Here, $g_X(x)$ is the usual Rician probability density function (pdf), $H(x)$ is the Heaviside step function, which enforces the notion that the probability of $X < 0$ is zero, I_0 is a modified Bessel function and Q_1 is the Marcum Q-function. Written this way, $f_X(x)$ is valid for all $x \in \mathbb{R}$.

The statistical moments of the Rician distribution include:

$$E[X] = \bar{X} = \sigma\sqrt{\frac{\pi}{2}} L_{1/2}\left(-\frac{S^2}{2\sigma^2}\right)$$

$$E[X^2] = S^2 + 2\sigma^2 \tag{2}$$

$$\mathrm{Var}(X) = E[X^2] - E[X]^2 = 2\sigma^2 + S^2 - \frac{\pi\sigma^2}{2} L_{1/2}^2\left(-\frac{S^2}{2\sigma^2}\right)$$

$E[\cdot]$ is a statistical expectation that is generally estimated from sample averages. The Laguerre polynomial $L_{1/2}$ has useful expansions for both small- and large-magnitude arguments:

$$L_{1/2}(-a) = 1 + \frac{a}{2} - \frac{a^2}{16} + \cdots, \quad (\text{small } a)$$

$$L_{1/2}(-a) = \frac{2\sqrt{a}}{\sqrt{\pi}} + \frac{1}{2\sqrt{\pi}\sqrt{a}} + \cdots, \quad (\text{large } a) \tag{3}$$

In the limit $S \to 0$, the Rician distribution reduces to the Rayleigh distribution and at signal-to-noise ratio $S/\sigma \geq 3$ the Rician distribution is very closely approximated as:

$$f_X(x) \approx Normal\left(S\left(1 + \frac{\sigma^2}{2S^2}\right), \sigma^2\left(1 - \frac{\sigma^2}{2S^2}\right)\right) \tag{4}$$

in accordance with Marshall and Verdun. Additional details about the probability distributions referenced here and their interrelationships can be found in the literature.[3]

The formalism involving $H(x)$ provides a convenient way to incorporate censoring (zeroing of data points to manage volume of data). To censor data, any x that is smaller than a threshold is set to zero. Typically, the threshold is set at $K\sigma$,[4] a multiple K of the time-domain

noise standard deviation. The fraction of the data that are censored can be obtained via integration:

$$P_0 = \int_{-\infty}^{K\sigma} f_X(x)dx = Q_1\left(\frac{S}{\sigma}, K\right) \tag{5}$$

and a generalized function $f(x)$ that describes the probability structure of censored data is:

$$f(x) = P_0\delta(x) + g_X(x)H(x - K\sigma) \tag{6}$$

where $\delta(x)$ is the Dirac delta function. This is not a proper probability density function as it combines a probability mass P_0 at zero with the Rician pdf for data that exceed the threshold. However, it can be treated as such when computing probabilities.[5] It is notable that given the thresholding parameter K, the fraction of censored data depends solely on the ratio of signal to noise.

The MRI solution can be mapped to the FTMS problem by recalling that the signal magnitude S_i of a mass peak for an ion of type *i* is proportional to the number of ions n_i of that type in the trap.

$$S_i = An_i \tag{7}$$

In addition, for replicate measurements n_i is not constant, but rather, is drawn from some discrete distribution $n_i \sim D(\theta)$ with probability mass function D and parameter set θ. As a result, the Rician probability function describes a distribution conditioned on a precise number of trapped ions n_i. The expected value of the mass-peak height is obtained by marginalizing over n_i as outlined in Equations (8).

$$\begin{gathered} E[X_i|n_i] = \int_{K\sigma}^{\infty} X_i f(An_i)\, dX_i \\ E[X_i] = \sum_{j=0}^{\infty} D(j|\theta)\ E[X_i|j] \end{gathered} \tag{8}$$

Similarly:

$$E[X_i^2|n_i] = \int_{K\sigma}^{\infty} X_i^2 f(An_i)\, dX_i$$

$$E[X_i^2] = \sum_{j=0}^{\infty} D(j|\theta)\ E[X_i^2|j] \qquad (9)$$

$$\text{Var}(X_i) = E[X_i^2] - E[X_i]^2$$

Note that the integrals reduce to the expressions in Equations (2) when the probability of censoring is very small. To summarize, the distribution of a single mass peak is given by a weighted sum of censored Rician distributions (Equation (6)) with weights given by the distribution D of the number of ions contributing to that peak. This distribution will be denoted WSoR. If the C-trap collects secondary ions at a constant rate λ_i for time t_c, ion accumulation is a Poisson process[6] and the number of trapped ions will be Poisson distributed, $n_i{\sim}Poisson(\lambda_i t_c)$.

Substituting Equation (7) for S into Equation (5), the Orbitrap detection limit for a given ion can be conveniently expressed in terms of the number of those ions in the trap. We take the detection limit to be the number of ions for which there is a 99.9% probability of observing a non-zero signal given the threshold. This corresponds approximately to the three-sigma confidence level for a Gaussian distribution and is found as the point where the Q-function takes on the value 0.001. K is a fixed, instrument-defined parameter. Consequently, the detection limit depends only on the ratio of the ions-to-signal scale factor A to the time-domain noise standard deviation σ. Taking values of A and $\bar{\sigma}$ for the silver depth profiles from Table 1 in the main text, this ratio is 1.5 independent of beam current implying a detection limit of 3.7 ions.

Statistics of OrbiSIMS ion generation and transfer

OrbiSIMS employs a primary ion beam to generate a stream of secondary ions whose distribution reflects the composition of the sampled material. To produce a single mass spectrum, ions are generated at a rate λ for time t_c and transferred to the Orbitrap analyser with efficiency τ. Secondary ion generation is a Poisson process. If λ is strictly constant, N_0, the total number of ions generated given λt_c, is itself Poisson-distributed: $N_0|\lambda t_c{\sim}Poisson(\lambda t_c)$. However, if the generation rate is subject to variation or noise, this process can be generalized. Allowing λt_c to vary according to some distribution with mean μ_λ and variance σ_λ^2, the unconditional distribution of N_0 is characterized by:

$$E[N_0] = E\big[E[N_0|\lambda t_c]\big] = E[\lambda t_c] = \mu_\lambda$$
$$\mathrm{Var}(N_0) = E[\mathrm{Var}(N_0|\lambda t_c)] + \mathrm{Var}(E[N_0|\lambda t_c]) = \mu_\lambda + \sigma_\lambda^2 \quad (10)$$

The variance of a Poisson random variable is equal to its mean. By allowing the ion generation rate to vary, the variance of N_0 increases by the variance of λt_c and becomes overdispersed with respect to Poisson. It will be convenient to rewrite the variance:

$$\mathrm{Var}(N_0) = \mu_\lambda + \left(\frac{\sigma_\lambda^2}{\mu_\lambda^2}\right)\mu_\lambda^2 = \mu_\lambda + R_\lambda^2\mu_\lambda^2 \quad (11)$$

Here, R_λ is the relative standard deviation attributed to overdispersion. Note that Eq. (11) predicts the total number of generated ions is a quadratic function of its mean. Overdispersion is often the dominant component of variance in mass spectral imaging techniques, such as MALDI, where there may be significant variation in sample-to-sample composition, hence ion generation rate. Over-dispersed Poisson data are frequently modeled as negative binomial. Summary statistics for N_0 drawn from:

$$N_0 \sim NegBin\left(\frac{1}{R_\lambda^2}, \frac{1}{1 + R_\lambda^2\mu_\lambda}\right) \quad (12)$$

are precisely those in Eqs. (10).

After the secondary ions have been generated, a system of ion optics directs the ions to the Orbitrap analyser. A fraction of the generated ions is trapped. If τ is the probability that a given ion is successfully transferred and trapped, transfer/trapping can be represented as a binomial thinning process.[7] The distribution of the number of ions trapped N conditioned on the number generated and the trapping probability is binomial:

$$Prob(N|N_0,\tau) \sim Binomial(N_0,\tau) \quad (13)$$

Marginalizing over N_0 yields:

$$E[N|\tau] = E\big[E[N|N_0,\tau]\big] = E[N_0\tau] = \tau\mu_\lambda$$
$$\mathrm{Var}(N|\tau) = E[Var(N|N_0,\tau)] + Var(E[N|N_0,\tau]) = \tau\mu_\lambda + \tau^2\sigma_\lambda^2 \quad (14)$$

For constant τ, the mean and variance of the number of trapped ions have values expected of scaled random numbers, namely, the mean is scaled by τ and the variance by τ^2. The thinning process retains the Poisson or negative binomial character of the input. In the negative binomial case:

$$N|\tau \sim NegBin\left(\frac{1}{R_\lambda^2}, \frac{1}{1+\tau R_\lambda^2 \mu_\lambda}\right) \tag{15}$$

As with ion generation rate, the transfer/trapping probability might vary due to instrumental fluctuations. Given τ distributed according to some distribution with mean μ_τ and σ_τ^2, the statistics for the number of trapped ions *N* is obtained by marginalizing over τ with the result:

$$\begin{gathered} E[N] = \mu_\lambda \mu_\tau = \mu_N \\ \mathrm{Var}(N) = \mu_N + \mu_N^2\left(\frac{\sigma_\lambda^2}{\mu_\lambda^2} + \frac{\sigma_\tau^2}{\mu_\tau^2}\right) = \mu_N + R_N^2 \mu_N^2 \\ N \sim NegBin\left(\frac{1}{R_N^2}, \frac{1}{1+R_N^2 \mu_N}\right) \end{gathered} \tag{16}$$

R_N is the relative standard deviation in the number of trapped ions in excess of the Poisson value and the latter expression neglects second-order terms in the variance. Note that Eqs. (16) have precisely the same form as Eqs. (14). Without additional information the ion generation and trapping processes cannot be individually identified. However, the OrbiSIMS has a very stable ion generation source, so it is not unlikely that variations in ion transfer or trapping contribute significantly to the observed overdispersion. Including it explicitly in the model provides a framework for addressing these questions in future experiments.

Second-order statistics of a mass spectrum

We have hitherto been concerned solely with variation in the total number of ions being generated and trapped without regard to their mass or identity. The Orbitrap mass analyzer partitions a mixture of N trapped ions into k bins defined by the frequency resolution. The distributions of the individual types of ions are conveniently modeled in terms of the multinomial distribution. Conditioned on fixed N, the properties of the multinomial distribution include:

$$\begin{gathered} \mathbf{n} = [n_1, n_2, \cdots, n_k]^T, \quad n_i \in \{0, \dots, N\}, \ i \in \{1, \dots, k\}, \ \sum_i n_i = N \\ E[\mathbf{n}|N] = N\boldsymbol{\pi} \\ \mathrm{Var}(\mathbf{n}|N) = N\boldsymbol{\pi}(1-\boldsymbol{\pi}) \\ \mathrm{Cov}(\mathbf{n}|N) = -N\left(\boldsymbol{\pi}\boldsymbol{\pi}^T - \mathrm{diag}(\boldsymbol{\pi})\right) \end{gathered} \tag{17}$$

Here, all vectors are taken to be column vectors and are denoted by boldface characters. Superscript T is the transpose operator and the diag operator converts a vector to a diagonal matrix. The k-vector $\boldsymbol{\pi}$ expresses the underlying probabilities that ions oscillating at the corresponding frequencies are present in the sampled material.

The multinomial distribution is inherently multivariate with non-zero covariances. In fact, the off-diagonal elements of the covariance matrix are strictly negative. This reflects the fact that for fixed N, more than the average number of ions of one type must be offset by reduction in the numbers of other ions. Marginalizing over N, the means and variances of specific ions are:

$$\begin{gathered} E[\boldsymbol{n}] = E\big[E[\mathbf{n}|N]\big] = \mu_N \boldsymbol{\pi} \\ \mathrm{Var}(\mathbf{n}) = E[\mathrm{Var}(\mathbf{n}|N)] + \mathrm{Var}(E[\mathbf{n}|N]) = E[\mathbf{n}] + R_N^2 E[\mathbf{n}]^2 \end{gathered} \tag{18}$$

with the squares computed elementwise. The covariance is a little more interesting. Using the conditional covariance formula,[8] the off-diagonal elements of the covariance matrix can be written:

$$\mathrm{Cov}(\mathbf{n}) = \left(\frac{\mathrm{Var}(N)}{\mu_N^2} - \frac{1}{\mu_N}\right)\mu_N^2\left(\boldsymbol{\pi}\boldsymbol{\pi}^T - \mathrm{diag}(\boldsymbol{\pi}^2)\right) \tag{19}$$

Recall that the variance of a Poisson random variable is equal to its mean. Thus, for Poisson *N*, the covariance vanishes, and the data appear uncorrelated. As *N* becomes overdispersed, the data become increasingly positively correlated. Recognizing that $1/\mu_N$ is the squared relative standard deviation of a Poisson N, the factor in parentheses containing it is seen to be R_N^2, the squared relative standard deviation attributable to overdispersion. The positive off-diagonal covariances can then be rewritten as:

$$\mathrm{Cov}(\mathbf{n}) = R_N^2\left(E[\mathbf{n}]E[\mathbf{n}]^T - diag(E[\mathbf{n}]^2)\right) \tag{20}$$

The next step in our development is to convert the number of ions to a mass spectrum $\mathbf{s}$. This is accomplished by applying Eq. (7) elementwise to **n**. For simple scaling, the expected statistics of the mass spectral signal are easily found to be:

$$\begin{gathered} E[\mathbf{s}] = AE[\mathbf{n}] = \boldsymbol{\mu} \\ \mathrm{Var}(\mathbf{s}) = A^2\mathrm{Var}(\mathbf{n}) = A\,E[\mathbf{s}] + R_N^2 E[\mathbf{s}]^2 \\ \mathrm{Cov}(\mathbf{s}) = diag(A\,E[\mathbf{s}]) + R_N^2 E[\mathbf{s}]E[\mathbf{s}]^T \end{gathered} \tag{21}$$

Here, $\mathrm{Cov}(\mathbf{s})$ is the full covariance matrix, the sum of the diagonal variance matrix with the hollow matrix of Eq. (20). These expressions account for the source-dependent variations of OrbiSIMS signal in the absence of detector noise. The variance is a quadratic function of the mean, and the off-diagonal elements of the covariance matrix comprise the outer product of the mean spectrum scaled by the square of the relative standard deviation, RSD, due to overdispersion.

A comprehensive model for OrbiSIMS data generation and noise that is appropriate to the full range of S/N can now be described. Since the distribution of data in the Orbitrap spectra is a weighted sum of Rician distributions, with weights derived from the distribution of the number of ions, then the negative binomial distribution governing the total number of ions in the trap together with the multinomial distribution describing mass separation allow the weights and requisite probabilities for the expectations in Eqs. (8) and (9) to be computed. Together with the instrument-defined thresholding parameter K, the data distribution, referred to as WSoR herein, is characterized in terms of four parameters: A and R_N that relate ion-counting statistics, and the intrinsic detector noise variances σ_W^2 and σ_F^2. These need to be estimated from experimental data. Values corresponding to the silver depth profiles are summarized in **Table 1** of the main text.

Taking examples from the silver depth profiles, single-ion signal distributions in the regime where detector noise is significant are provided in **Supplementary Figure 1**. Here, histograms of the signal intensities of three different ions are compared at each of the three beam currents. The mean number of trapped ions ranges from 0.48 to 9.48 across these examples and agreement is acceptable in all cases. In addition, the relationship between the variance and mean of a mass spectral peak, which has played a prominent role in our analysis, can be predicted from the WSoR distribution. **Supplementary Figure 2a-c** compares the observed data (deflated to remove the unmodeled cluster-size anticorrelations as described in **Supplementary Note 2: Experimental details for silver measurements**) with WSoR predictions at 100 logarithmically spaced points assuming n_i is drawn from the negative binomial distribution. Here, the mean value of the detector-noise standard deviation $\bar{\sigma}$ is used since low-intensity peaks may span the entire mass range. Excellent agreement is achieved for all three beam currents. The one outlying point in the 100-pA data corresponds to Cs^+, which will be discussed later (**Supplementary Note 7: Description of data scaling and transformations for multivariate analysis and comparison using silver datasets**). It will also prove useful for the forthcoming discussion about data scaling to consider Poisson-distributed n_i. In this case, the effects of overdispersion are excluded and the proper comparison is with the PFA estimate of the variance, which only captures the Poisson-like contribution. These comparisons are shown in **Supplementary Figure 2**d-f, and again, agreement is excellent. It is notable, in this case, that the WSoR distribution has only two adjustable parameters.

It should be noted that for large numbers of overdispersed ions, the number of terms in the summation of Eqs. (8) and (9) can be very large. To facilitate computations in the high-signal case, the negative binomial distribution can be approximated by a normal distribution having

mean and variance given by Eqs. (16). Furthermore, we can make use of the normal approximation to the Rician distribution provided in Eq. (4). This implies that the distribution of the signal s will be Gaussian. Using the laws of total expectation and total variance, the expected value of s and its variance are found to be:

$$E[s] = A\mu_N + \frac{\sigma^2}{2A} E\left[\frac{1}{n}\right]$$
$$\mathrm{Var}(s) = 2\sigma^2 + A^2 E[n^2] - E[s]^2$$

The expectations involving n can be easily computed assuming there is no probability that $n = 0$. For the silver datasets described in this paper, $\mu_n \gtrsim 20$ satisfy these conditions. Note that the WSoR distribution for the $^{107}Ag^{109}AgH_2O^+$ peak in the 200-pA dataset with $\mu_n = 9.48$ is already developing a Gaussian appearance as shown in **Supplementary Figure 1** .

Modeling multiple mass spectra and Probabilistic Factor Analysis

The foregoing development considers the multivariate mean and covariance of a single mass spectrum. In applications such as mass spectral imaging, many mass spectra are acquired, not all of which sample analyte of the same composition. Typically, chemical applications assume that Beer's Law-like behavior prevails where a given spectrum is a linear combination of a limited number of component spectra weighted by their respective abundances in the sample and contaminated with additive noise:

$$\mathrm{s} = \mathbf{Wz} + \mathbf{s}_E \tag{22}$$

For a k-channel spectrum **s** composed of q component spectra, **W** is a $k \times q$ matrix whose columns are the component spectra and **z** is a q-vector containing the abundances. We will consider a slightly different probabilistic model that takes **z** to be a zero-mean standard Gaussian random variable and isolates the (noise-free) spectral mean $\boldsymbol{\mu}$ as a separate parameter. The conditional probability of the spectrum given **z** is assumed to be:

$$\mathbf{s}|\mathbf{z} \sim Normal(\mathbf{Wz} + \boldsymbol{\mu}, \boldsymbol{\Sigma}) \tag{23}$$

where $\boldsymbol{\Sigma}$ is a *diagonal* covariance matrix that captures variance unique to each variable. For correlated variables such as isotopes of silver, the unique variance is noise. Marginalizing over $\mathbf{z}$ yields[9]:

$$\mathbf{s} \sim Normal(\boldsymbol{\mu}, \mathbf{WW}^T + \boldsymbol{\Sigma}) \tag{24}$$

Multiple spectra can be collected as rows of a data matrix **S**, in which case model Eq. (23) can be referred to the matrix equation:

$$\mathbf{S} = \mathbf{Z}\mathbf{W}^T + \mathbf{1}_m\boldsymbol{\mu}^T + \mathbf{S}_E \tag{25}$$

Given m spectra in the matrix $\mathbf{S}$, $\mathbf{1}_m$ is an m-vector of ones. Probabilistic Factor Analysis[9] PFA is a machine learning algorithm that can estimate the parameters $\boldsymbol{\mu}$, $\mathbf{Z}$, $\mathbf{W}$ and $\boldsymbol{\Sigma}$ given the probabilistic model expressed in Eq. (24). It should be noted that if $\boldsymbol{\Sigma}$ is taken to be the identity matrix (i.e., homoscedastic data), PFA is a probabilistic algorithm for computing PCA[10,11], the standard Principal Components Analysis factorization. In other words, PCA implicitly makes the same normal distribution assumption made explicit in PFA. PFA results presented in this paper can be computed with MATLAB code available on the Mathworks File Exchange.[12] Additionally, we have rotated PFA factors to the PCA basis using a method described previously.[13] This orthogonalizes the components and orders them in terms of significance enabling direct comparisons across multiple datasets and methods.

It is interesting to consider PFA in the context of the experimental data of this work where all spectra are nominally replicates with the same mean spectrum. Thus, **Z** and **W** should vanish in the ideal case with uncorrelated noise $\mathbf{S}_E$ drawn row-wise from $s_E \sim Normal(\mathbf{0}, \boldsymbol{\Sigma})$. Under the standard assumption that signal and noise are uncorrelated, the expected outer product of a spectrum is:

$$E[\mathbf{s}\mathbf{s}^{\boldsymbol{t}}] = (1 + R_N^2)\boldsymbol{\mu}\boldsymbol{\mu}^{\boldsymbol{T}} + diag(A\boldsymbol{\mu}) \tag{26}$$

where we have substituted Eq. (21) for the error covariance of an OrbiSIMS spectrum. This equation predicts that the diagonal of $\boldsymbol{\Sigma}$ estimated by PFA should be a scaled version of the mean spectrum. In other words, PFA should estimate the Poisson-like component of the covariance. Additionally, the mean spectrum is the only spectral vector in the model suggesting that we should expect only a single significant component in the PFA-based model of the mass spectral data matrix. Finally, it should be noted that if there is real depth variation for any component, or there are experimental artifacts that influence different types of ions differently, the variance is additive, and the additional components will be described in **Z** and **W**. In the latter case, the artifactual effects can be approximately eliminated from the data via deflation:

$$\mathbf{S}_{Deflated} = \mathbf{S} - \mathbf{Z}\mathbf{W}^T \tag{27}$$

Adaptively controlling the number of ions

The fundamental assumption underlying the linearity between the measured image current and the number of ions in the Orbitrap analyser is that all trapped ions oscillate independently of one another. This assumption is violated when nonlinearities, such as

space-charge effects, become important. One strategy for avoiding these complications is to limit the number of ions allowed into the Orbitrap analyser. The Orbitrap MS includes an Automatic Gain Control system (AGC) that can adjust the number of ions by varying the period over which the C-trap is filled, i.e., t_c in Eq. (10). The OrbiSIMS takes a slightly different approach with its Adaptive Ion Injection System (AIIS).

To produce a single mass spectrum in the depth profiles discussed here, the primary ion beam is rastered across a predefined field of view (FoV) and ions from all pixels are collected in the C-trap. If, based on prior mass spectra, the predicted number of ions exceeds the maximum number allowed, AIIS reduces the number of pixels sampled in the FoV per injection. This reduction can be parameterized in terms of a "suppression" factor ψ. For instance, if $\psi = 2$ half the pixels in the FoV are sampled. AIIS can be operated in two limiting modes: "fast" mode, with a single injection per FoV discarding secondary ions that would lead to saturation and "sensitive" mode, which samples all pixels over the course of $N_{inject} = \psi$ injections. To put all mass spectra on a common basis, the reduced-intensity mass spectra are upscaled by ψ in "fast" mode. AIIS can be accommodated in the probabilistic model by replacing the scale factor A with $A\psi$, taking expectations $E[n/\psi]$ and recognizing variance is reduced by N_{inject} for N_{inject} nominally replicate measurements. With these modifications, Eqs. (21) become:

$$
\begin{aligned}
E[\mathbf{s}] &= A\psi E\left[\frac{\mathbf{n}}{\psi}\right] \\
\mathrm{Var}(\mathbf{s}) &= \frac{\psi}{N_{inject}}\left(A\,E[\mathbf{s}] + \frac{1}{\psi}R_N^2 E[\mathbf{s}]^2\right) \\
\mathrm{Cov}(\mathbf{s}) &= \frac{\psi}{N_{inject}}\left(\mathrm{diag}(A\,E[\mathbf{s}]) + \frac{1}{\psi}R_N^2 E[\mathbf{s}]E[\mathbf{s}]^T\right)
\end{aligned}
\quad (28)
$$

These equations are appropriate for a single spectrum in the case that detector noise is negligible. At low signal levels, S/N is reduced in both AIIS modes. The noise is directly multiplied by ψ in "fast" mode, increasing the threshold by the same amount. "Sensitive" mode adds together noise from each of the ψ injections. Typical applications acquire multiple mass spectra and ψ can vary from spectrum to spectrum. In "fast" mode, individual values of ψ comprise the diagonal of a diagonal matrix $\mathbf{\Psi}$, which can be used to scale the variances. This has the same form as a row-wise noise covariance matrix[14], which may be useful for scaling the data in multivariate analysis.

Alternative description of ion counting and transferring statistics

At signal levels where detector noise is negligible, variations in the total number of ions trapped and how they are separated in the mass analyzer dominate the noise. In the

foregoing, the total number of trapped ions was modeled as a negative binomial random variable to account for the observed overdispersion with respect to Poisson statistics, and the partitioning of the trapped ions into mass bins was governed by the multinomial distribution. The following discussion details how the negative binomial distribution arises naturally from models of ion generation and transfer rates and describes a structured approach for incorporating different or additional sources of variation. We will also provide an alternative point of view showing how the same compound negative binomial/multinomial distribution is produced by independent inhomogeneous Poisson processes run in parallel.

Producing a mass spectrum entails several steps, each of which introduces an element of random variation into the result. Counting statistics, the inherent variability of counting the number of randomly occurring events in a fixed time interval, is the primary source of variation. In the present case, at least three counting processes can be identified. Ions to be trapped and analyzed are first generated and subsequently transferred to the Orbitrap MS. The total number of ions generated N_0 is a random quantity that depends on the generation rate λ, and the total number trapped N varies randomly with both N_0 and the efficiency τ of their transfer to the Orbitrap analyser. Assuming N comprises k distinct types of ions, the mass analyzer partitions those ions into k mass bins with variability that depends on the probabilities π_i that ions of type i are present in the trapped mixture. It will be convenient to represent the partitioned set of ions by the k-vector $\mathbf{n}$ with corresponding probability vector $\boldsymbol{\pi}$. Vector quantities are denoted by boldface characters and taken to be column vectors.

Besides these counting-based sources of variation, our model allows for possible spectrum-to-spectrum fluctuations in the ion generation rate and transfer efficiency. Such variability gives rise to overdispersion of the data with respect to Poisson statistics. Overdispersion is often the dominant component of variance in mass spectral imaging techniques, such as MALDI, where there may be significant variation in sample-to-sample composition, hence ion generation rate. Additionally, the OrbiSIMS has a very stable ion generation source, so it is not unlikely that variations in ion transfer or trapping contribute significantly to observed overdispersion.

Ultimately, we are interested in the expected value, variance and covariance of the random vector $\mathbf{n}$ given its dependence on the four additional random variables N, N_0, λ and τ. Evaluating the statistics requires making several distributional assumptions. These are summarized in the Table. Three conditional distributions govern discrete counts. As noted above, ion generation is a Poisson process and the number of ions generated N_0 conditioned on the generation rate λ is Poisson-distributed. After the secondary ions have been generated, a system of ion optics directs the ions to the Orbitrap MS. Only a small

fraction of the ions is trapped. If τ is the probability that a given ion is successfully transferred and trapped, transfer/trapping can be represented as a binomial thinning process[7] and the total number of trapped ions N conditioned on N_0 and τ is binomially distributed. Finally, the total number of trapped ions N is divided among k mass peaks in the mass analyzer. The individual ion counts $\mathbf{n}$ given underlying relative abundances $\boldsymbol{\pi}$ in the sampled material follow the multinomial distribution, which satisfies the conditions $\mathbf{1}_k^T\boldsymbol{\pi} = 1$ and $\mathbf{1}_k^T\mathbf{n} = N$. Here, superscript T is the transpose operator and $\mathbf{1}_k$ is a k-vector of ones.

Two additional distributions are needed to describe the ion generation rate λ and trapping efficiency τ. These are characterized by mean values, μ_λ and μ_τ, respectively. Additionally, these parameters are allowed to vary from spectrum to spectrum with respective variances $\sigma_\lambda^2 = \mu_\lambda^2 R_\lambda^2$ and $\sigma_\tau^2 = \mu_\tau^2 R_\tau^2$, which are conveniently parameterized in terms of the relative standard deviations R_λ and R_τ. The ion generation rate is inherently positive and can be reasonably modeled as a gamma-distributed random variable. Assuming:

$$\lambda \sim Gamma\left(\frac{1}{R_\lambda^2}, R_\lambda^2 \mu_\lambda\right) \tag{29}$$

yields λ with the desired mean and variance. Similarly, τ is the probability that a generated ion will be successfully transferred to the Orbitrap MS and trapped. The beta distribution, which has support (0, 1), is useful for modelling probabilities. With the following parameter choices, $E[\tau] = \mu_\tau$ and $\mathrm{Var}(\tau) = \mu_\tau^2 R_\tau^2$:

$$\begin{gathered} \tau \sim Beta(\alpha, \beta) \\ \alpha = \frac{1 - \mu_\tau(1 + R_\tau^2)}{R_\tau^2}, \qquad \beta = \alpha\frac{1 - \mu_\tau}{\mu_\tau} \end{gathered} \tag{30}$$

Under conditions typical of the silver depth profile experiments, the gamma and beta distributions very closely approximate the normal distribution. Thus, these distributions effectively model ion generation rate and transfer efficiency exhibiting Gaussian variations. It should be noted that compounding a Poisson distribution with a gamma-distributed rate parameter yields the negative binomial distribution, and that compounding the binomial distribution with a beta-distributed probability parameter gives the beta-binomial distribution. These distributions are summarized in the Table. Whilst we assume in this paper that λ and τ are scalar quantities, they could, in principle, be generalized to vectors to accommodate potential mass dependencies.

The expected value of $\mathbf{n}$ is easily computed from the law of iterated expectations realizing that N_0 and τ are independent, and that given N, $\mathbf{n}$ is conditionally independent of the other random variables:

$$E[\mathbf{n}] = E[E(\boldsymbol{n}|N)] = E[N\boldsymbol{\pi}] = E[E[E[N|N_0,\tau]|\tau]|N_0]\boldsymbol{\pi} = \mu_\lambda\mu_\tau\boldsymbol{\pi} \tag{31}$$

Similarly, the variance of $\mathbf{n}$ can be obtained from a generalization of the law of total variance[15]:

$$\begin{aligned}\mathrm{Var}(\mathbf{n}) = {} & E[\mathrm{Var}(\mathbf{n}|\tau,\lambda,N_0,N)] + E[\mathrm{Var}(E[\mathbf{n}|\tau,\lambda,N_0,N]|\tau,\lambda,N_0)] \\ & + E[\mathrm{Var}(E[\mathbf{n}|\tau,\lambda,N_0]|\tau,\lambda)] + E[\mathrm{Var}(E[\mathbf{n}|\tau,\lambda]|\tau)] + \mathrm{Var}(E[\mathbf{n}|\tau])\end{aligned} \tag{32}$$

The properties of conditional expected values together with relationships in the Table can be used to evaluate this expression with the result:

$$\mathrm{Var}(\mathbf{n}) = \left(\mu_\lambda\mu_\tau + \mu_\lambda^2\mu_\tau^2(R_\lambda^2 + R_\tau^2 + R_\lambda^2R_\tau^2)\right)\boldsymbol{\pi}^2 \tag{33}$$

where the vector square is performed elementwise. It is important to note the mean and variance expressions are invariant to exchanging subscripts. In other words, without additional information the ion generation and trapping processes cannot be individually identified. We have included both terms explicitly in the model to provide a framework for analyzing future experiments where side information is available. Making the definitions: $\mu_N \equiv \mu_\lambda\mu_\tau$ and $R_N^2 \equiv R_\lambda^2 + R_\tau^2 + R_\lambda^2R_\tau^2$ yields:

$$\begin{aligned}E[\mathbf{n}] &= \mu_N\boldsymbol{\pi} \\ \mathrm{Var}(\mathbf{n}) &= (\mu_N + \mu_N^2R_N^2)\boldsymbol{\pi}^2\end{aligned} \tag{34}$$

Comparison with the table also shows that μ_N and $\mu_N + \mu_N^2R_N^2$ have precisely the same forms as the mean and variance of a negative binomial random variable. This provides a justification for drawing N from a negative binomial distribution in simulations. Finally, when parameterized this way, the variance of N is seen to consist of a Poisson component equal to its mean and an overdispersion component whose magnitude increases with its relative standard deviation.

As noted earlier, $\mathbf{n}|N \sim Multinomial(N, \boldsymbol{\pi})$. The multinomial distribution is inherently multivariate with non-zero covariances. In fact, the off-diagonal elements of the covariance matrix are strictly negative reflecting the fact that for fixed N more than the average number of ions of one type must be offset by reduction in the numbers of other types of ions:

$$\mathrm{Cov}(\mathbf{n}|N) = -N\left(\boldsymbol{\pi}\boldsymbol{\pi}^T - \mathrm{diag}(\boldsymbol{\pi})\right) \tag{35}$$

Here, the diag operator converts a vector to a diagonal matrix. Using the conditional covariance formula[8], the off-diagonal elements of the covariance matrix of $\mathbf{n}$ can be written:

$$\mathrm{Cov}(\mathbf{n}) = \left(\frac{\mathrm{Var}(N)}{\mu_N^2} - \frac{1}{\mu_N}\right)\mu_N^2\left(\boldsymbol{\pi}\boldsymbol{\pi}^T - \mathrm{diag}(\boldsymbol{\pi}^2)\right) \tag{36}$$

Recall that the variance of a Poisson random variable is equal to its mean. Thus, for Poisson N, the covariance vanishes, and the data appear uncorrelated. As N becomes over-dispersed, the data becomes increasingly positively correlated. Recognizing that $1/\mu_N$ is the squared relative standard deviation of Poisson N, the factor in parentheses containing it equals R_N^2, the squared relative standard deviation attributable to overdispersion. The covariance matrix can then be rewritten as:

$$\mathrm{Cov}(\mathbf{n}) = \mu_N \mathrm{diag}(\boldsymbol{\pi}^2) + R_N^2 \mu_N^2 \boldsymbol{\pi}\boldsymbol{\pi}^T \tag{37}$$

Here, Cov($\mathbf{n}$) is the full covariance matrix including the variance along the diagonal.

It is interesting to note that precisely the same mean, variance and covariance can be obtained by compounding the multinomial distribution with a negative binomial N. The resulting distribution is the negative multinomial[16] as shown in the Table. The same negative multinomial distribution also describes k independent Poisson processes run in parallel[17] with the rate parameter at each time step drawn from the gamma distribution Eq (29). Given μ_N, R_N and $\boldsymbol{\pi}$, an m-sample ion-count matrix $\mathbf{N}$ can be simulated with two lines of Matlab pseudocode:

$$\mathbf{G} = \mathrm{gamrnd}(1/R_N^2\,, \mu_N * R_N^2, m, 1)$$
$$\mathbf{N} = \mathrm{poissrnd}(\mathbf{G} * \boldsymbol{\pi}^T)$$

In the absence of detector noise, a mass spectrum $\mathbf{s}$ is obtained by multiplying the ion-count vector $\mathbf{n}$ by the counts-to-signal scale factor A. The mean, variance and covariance of the mass spectra are simply obtained as in Eqs. (21) by replacing μ_N with $\mu_S = A\mu_N$ and recognizing that $R_S = R_N$. These expressions account for the source-dependent variations of OrbiSIMS signal when detector noise is negligible. The variance is a quadratic function of the mean spectrum, and the off-diagonal elements of the covariance matrix comprise the outer product of the mean spectrum scaled by the square of the relative standard deviation arising from overdispersion.

Supplementary Table 1

Probability	Distribution	Mean	Variance
$\mathbf{n}\|N$	$Multinomial(N, \boldsymbol{\pi})$	$N\boldsymbol{\pi}$	$N\boldsymbol{\pi}(\mathbf{1}-\boldsymbol{\pi})$
$N\|N_0, \tau$	$Binomial(N_0, \tau)$	$N_0\tau$	$N_0\tau(1-\tau)$
$N_0\|\lambda$	$Poisson(\lambda)$	λ	λ
λ	$Gamma\left(\frac{1}{R_\lambda^2}, \mu_\lambda R_\lambda^2\right)$	μ_λ	$\mu_\lambda^2 R_\lambda^2$
τ	$Beta\left(\alpha = \frac{1-\mu_\tau(1+R_\tau^2)}{R_\tau^2}, \beta = \frac{\alpha(1-\mu_\tau)}{\mu_\tau}\right)$	μ_τ	$\mu_\tau^2 R_\tau^2$
N_0	$NegativeBinomial\left(\frac{1}{R_\lambda^2}, \frac{1}{1+\mu_\lambda R_\lambda^2}\right)$	μ_λ	$\mu_\lambda + \mu_\lambda^2 R_\lambda^2$
$N\|N_0$	$BetaBinomial(N_0, \alpha, \beta)$	$N_0\mu_\tau$	$N_0\mu_\tau(1-\mu_\tau)$ $+ N_0(N_0-1)\mu_\tau^2 R_\tau^2$
$\mathbf{n}$ with $N \sim NegBin$	$NegativeMultinomial\left(\frac{1}{R_N^2}, \frac{1}{1+\mu_N R_N^2}[\boldsymbol{\pi}; 1]\right)$	$\mu_N\boldsymbol{\pi}$	$\mu_N\boldsymbol{\pi} + \mu_N^2 R_N^2 \boldsymbol{\pi}^2$

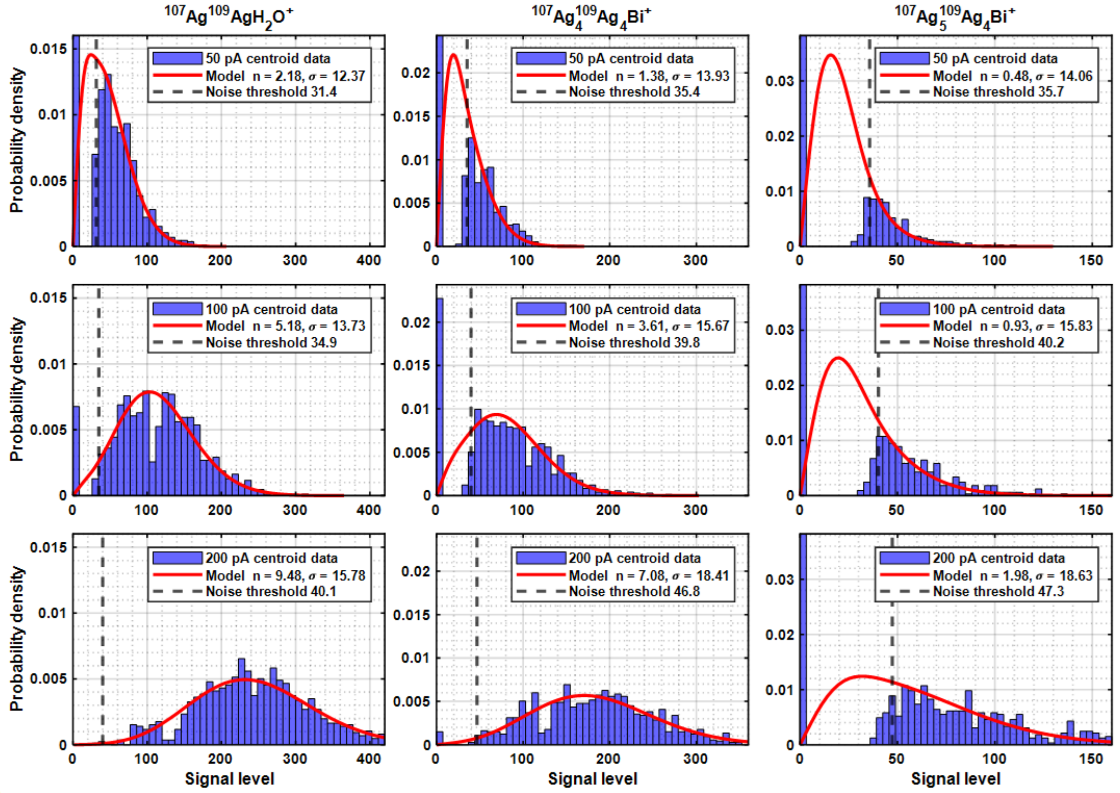

Supplementary Figure 1: Comparison of the histograms of three different ions at each of the three beam currents with the WSoR model predictions. The number of trapped ions range from 0.48 to 9.48

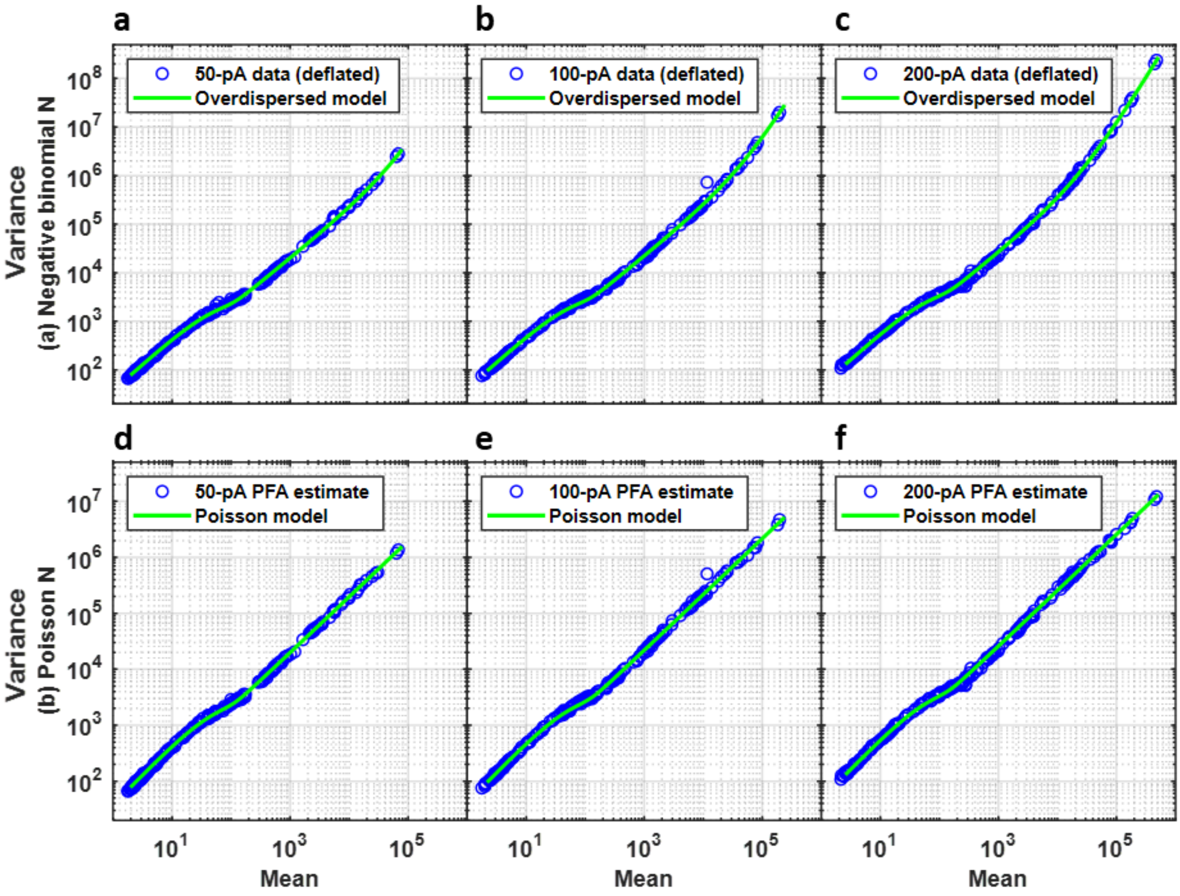

Supplementary Figure 2. **a-c** Comparison of the observed data (deflated to remove the unmodeled cluster-size anticorrelations) with WSoR predictions at 100 logarithmically spaced points assuming n_i is drawn from the negative binomial distribution. **d-f** Comparison between PFA estimate (that excludes overdispersion) and Poisson-like contribution of the model.

Supplementary Note 2: Extended experimental details for silver measurements

Datasets were acquired using an OrbiSIMS instrument (Hybrid SIMS, IONTOF GmbH). Depth profiles were obtained in a single beam mode using a 30 keV Bi_n^+ ion beam from a liquid metal ion source. The average primary ion beam currents were 50 pA, 100 pA and 200 pA. The beam was set to scan an area of 320 μm × 320 μm of which secondary ions were collected from the central 200 μm × 200 μm of the crater. 70 x 70 pixels were used per scan, which resulted in a single injection per scan. The analyser extraction voltage was set at 2000 V The Orbitrap mass analyser was operated with 512 ms injection time and 512 ms transient time for a mass resolving power of 240,000 at m/z 200. A total of 1000 scans were acquired for each beam current. For all OrbiSIMS analyses, the sample target potential was set to 57.5 V and the He collision cell pressure was kept at 0.12 bar in high collisional cooling

mode. **Supplementary Figure 3** shows a comparison between the beam target current stability (0.4 % RSD measured at a faraday cup for an arbitrary current) and the $^{107}Ag_1^+$ variation across 1000 scans (RSD 8.9%), which shows that the source is stable enough for the noise estimation described in the main text.

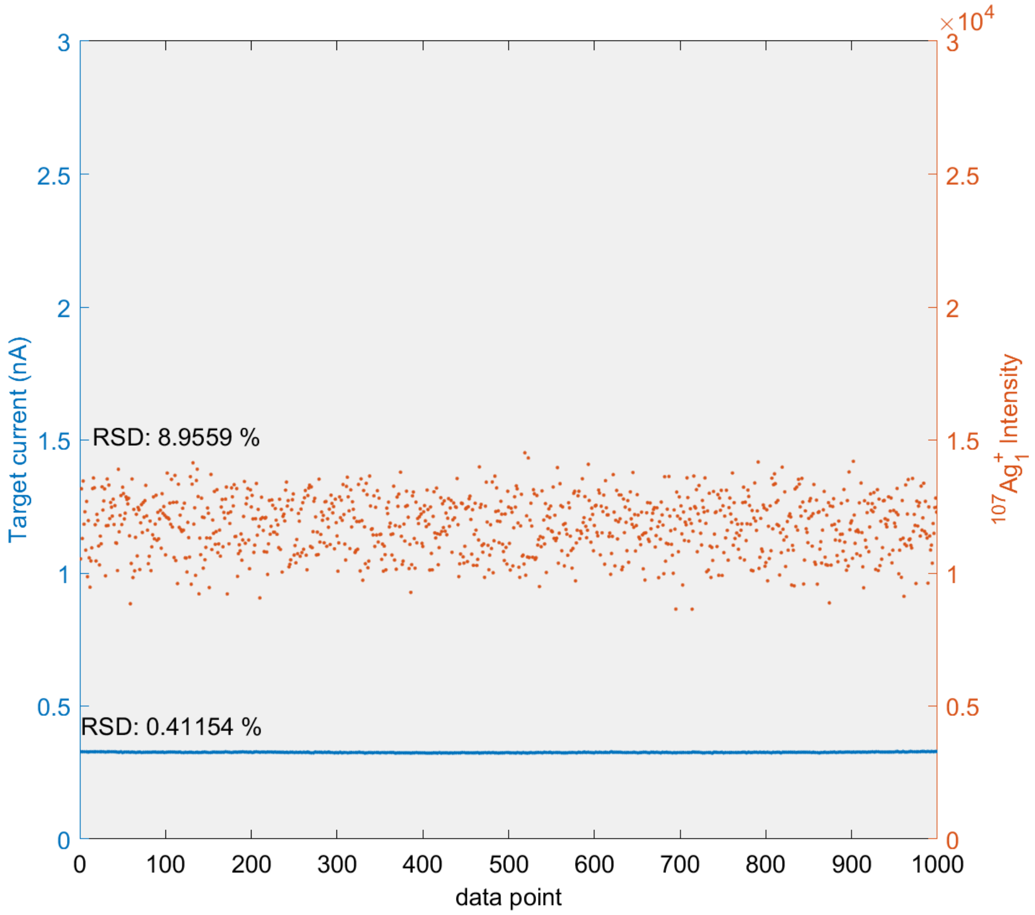

Supplementary Figure 3: Beam current stability (blue) and $^{107}Ag_1^+$ signal across 1000 scans (orange).

Supplementary Note 3: Further description of measured silver signal and unmodeled signal variations

In our probabilistic model, neither the mass of an ion nor its chemical identity influences the variance-to-mean relationship. Close examination of the variance-*vs*-mean plots for the present data suggests that this is not the case. **Supplementary Figure 4a** shows that small clusters, Ag_3^+ and smaller, have relatively less variance than the larger clusters in the 200-pA dataset. A specific example is given in **Supplementary Figure 4b**, which compares the distribution of a single isotope of Ag_3^+ with one of Ag_5^+ that has essentially the same mean intensity. The two distributions are expected to be the same, but clearly Ag_5^+ is both broader and appears to have a different shape. PFA (rotated to PCA) was used to isolate the

component giving rise to the broadening, which was observed in all three datasets. PC 2 is compared for the three beam currents in **Supplementary Figure 4c**. Whilst the details differ slightly, all three show anticorrelation of the small and large clusters. The physical origin of this difference is unknown. However, it is important to note that whilst this component is clearly present, it is a very small contribution to the overall variance in the data. In terms of variance captured by PCA, PC 2 accounts for only 0.02% of the total variance in the 200-pA dataset. After deflating the 200-pA dataset by the 2nd PC, the distributions of the Ag_3^+ and Ag_5^+ isotopes substantially coincide in **Supplementary Figure 4b**, and the variance vs mean plot collapses to a single curve as shown in **Supplementary Figure 4d**.

Another observation is that the experimental data are correlated at high signal level, although there is no mechanism in the basic noise model outlined by Marshall and Verdun to correlate the noise. The covariance matrix of the 200-pA dataset is shown in **Supplementary Figure 4e**, with the mean spectrum given on the margins. Note that the covariance matrix resembles a scaled version of the outer product of the mean spectrum. This suggests that correlation originates from all ions varying in tandem. The variance due to correlated noise adds to the Poisson random variation expected of ions acting independently resulting in the observed overdispersion.

In addition to isolating the source of excess variation in the larger silver clusters, PFA provides an estimate of the variance unique to each mass peak. **Supplementary Figure 4f** compares these estimates with the observed variances for the 200-pA dataset over the full intensity range. For mean intensities less than 1000, before overdispersion starts to become significant, the two coincide. Beyond that point, the estimates of the uncorrelated variance continue to track the slope-1 line characteristic of scaled Poisson data whilst the observed variances diverge. This helps to validate the basic decomposition of variance into uncorrelated Poisson-like and correlated overdispersion contributions inherent in the probabilistic model.

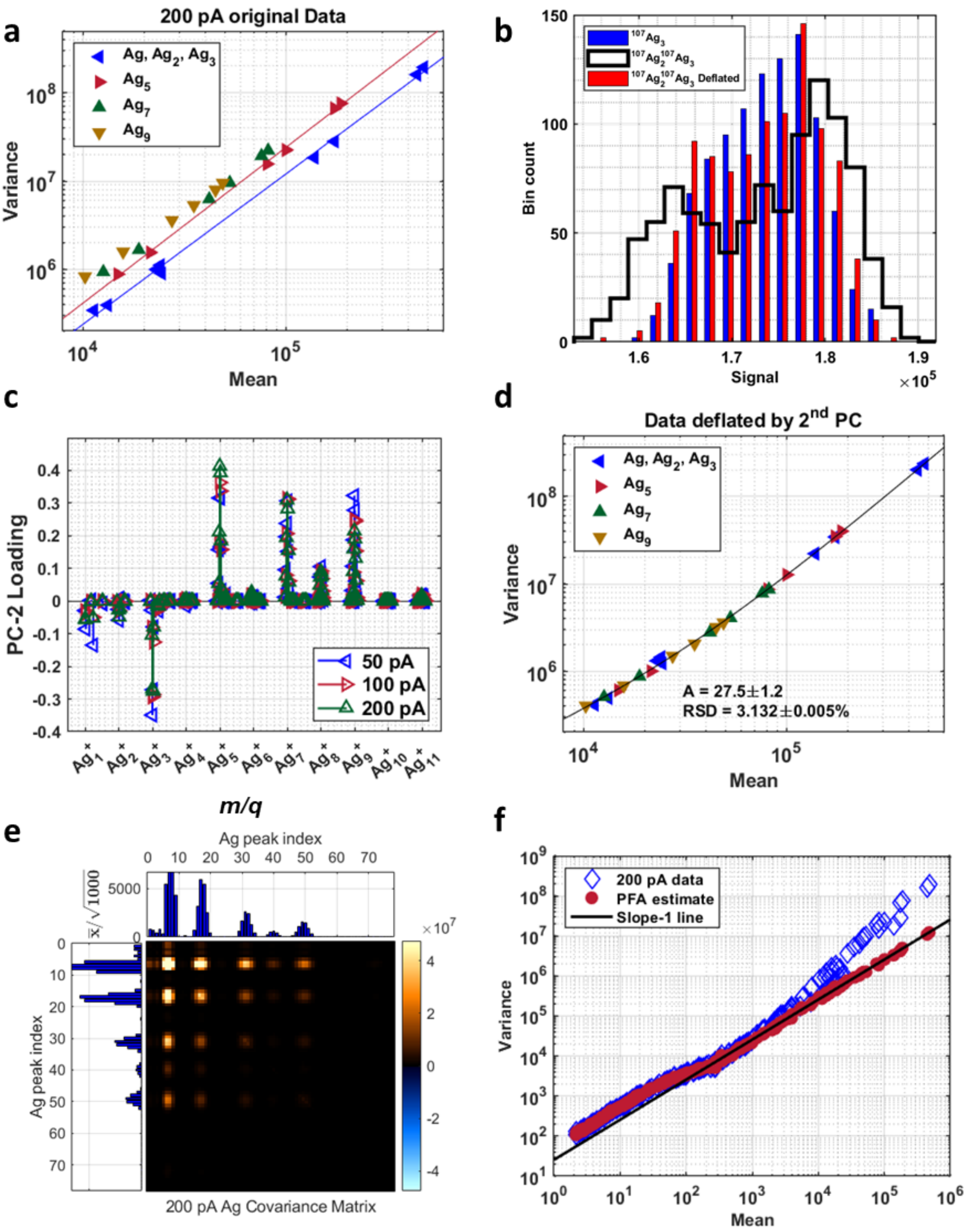

Supplementary Figure 4: a Variance and mean relationship for the 200-pA dataset in the high-signal regime. **b** Histograms for $^{107}Ag_3^+$ and $^{107}Ag_2{}^{109}Ag_3^+$ before and after deflation by PC 2. **c** Loadings of PC 2 showing anti-correlation between high and low-mass signal. **d** Data from **a** after deflation by PC 2. **e** Covariance of the silver peaks for the 200-pA dataset. The image has been clipped at 20% of the maximum intensity to improve contrast. The corresponding mean spectra are plotted on the margins **f** variance-mean relationships in the 200-pA dataset

There is a series of additional features in the data that are not modelled. Several of these are illustrated in **Supplementary Figure 5** and descriptions of panels **a – h** are provided below:

a) The combination of apodization with a Hann window and triple-zero padding creates additional frequency components in the Fourier transform resulting in spectral leakage.

b) The Orbitrap centroiding algorithm defines a peak as three consecutive points that exceed the noise threshold. Consequently, each lobe in the window frequency response in a) will be described by a single centroid. This results in a series of side-band peaks separated by four points as illustrated for a simulation of the main isotope peak of the 200-pA Ag_3^+ dataset. The series decays quickly to the noise level. These are the “exceptional” peaks that can be excluded from the OrbiSIMS output.

c) The full intensity range of the 200-pA Ag_3^+ cluster is provided for context. The spectrum looks “clean” at this scale.

d) Observed spectral leakage for the same isotope simulated in b). The structure is more complicated due, possibly, to a combination of eFT processing and other interferences. Notably, the decay rate is similar to the simulation when distant from the main peak.

e) Many extraneous peaks are found near the high-intensity peaks. The data have been binned by a factor of four to remove the discrete spectral leakage peaks induced by zero-padding and centroiding the data.

f) An expanded view of the mass spectrum near a large ion peak shows that the extraneous peaks are sidebands symmetrically placed about the main peak. The frequency intervals between sidebands are independent of mass. These may be due to interferences including, for instance, line frequency.

g) The noise baseline for the 200-pA data has complicated structure over a relatively broad mass range near Ag_3^+, the most intense ion cluster. The physical origin of this behavior is unknown. Besides variation in baseline intensity, the noise is highly correlated in this spectral region.

h) The 50-pA and 100-pA datasets show the same extraneous peaks at the same locations as the 200-pA dataset. Generally, the side-band intensities scale approximately linear with intensity of the main peak. The complicated noise baseline structure shown in f) is not noticeable in the 50-pA dataset and has much reduced intensity at 100 pA. Whatever the cause of the baseline anomalies in the 200-pA data, the effect increases more quickly than linear with beam current.

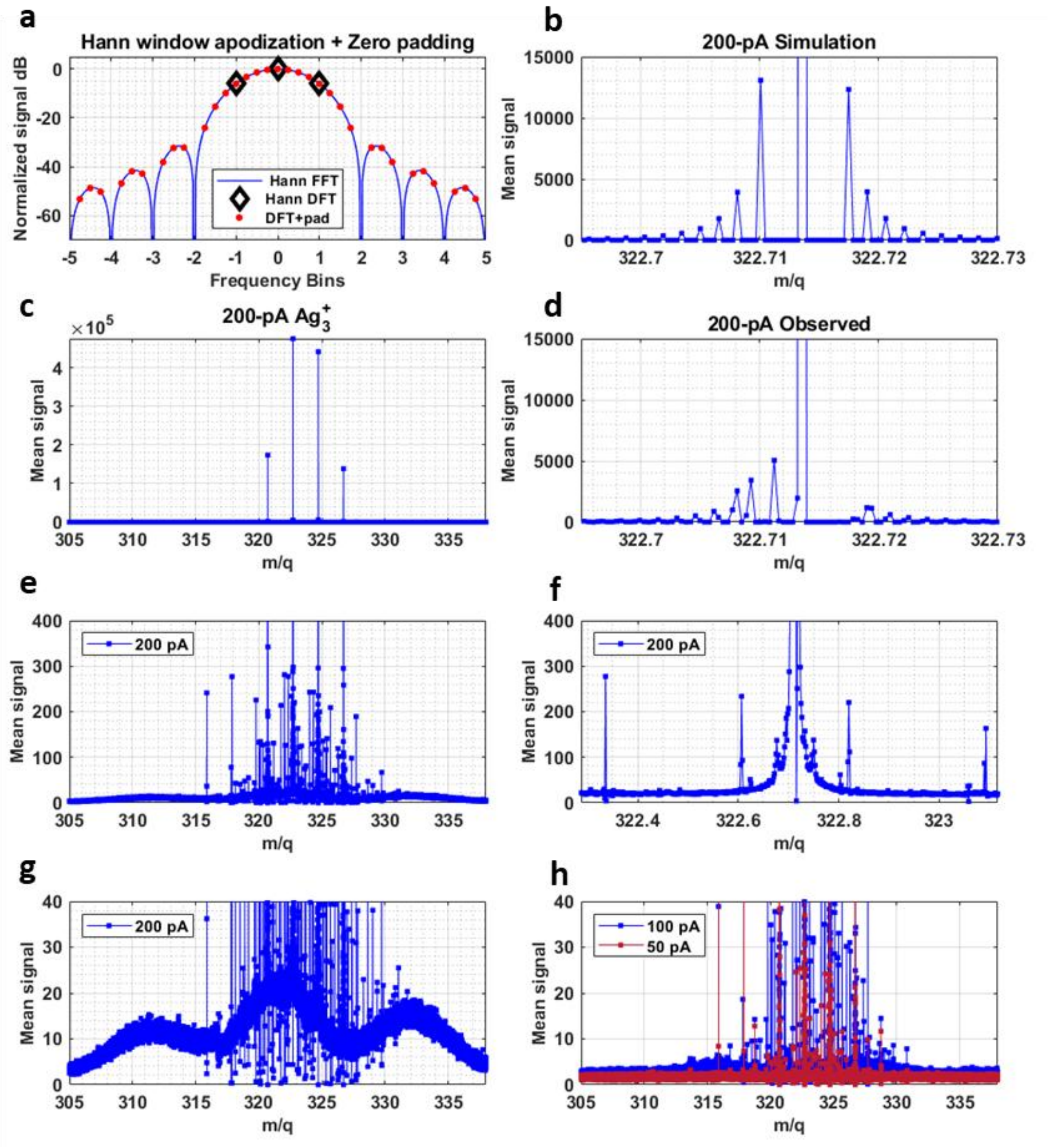

Supplementary Figure 5: Additional features identified in Orbitrap data.

It was noted in the main text and illustrated in **Supplementary Figure 5g**, that the baseline signal levels near high-intensity spectral peaks do not conform to the probabilistic model developed in this paper. Some specific examples of deviations that are not taken into account follow. These are simply observations; we make no attempt to explain the origins of these behaviors. **Supplementary Figure 6a** overlays the mean spectra for the three different beam currents in the vicinity of the 4 isotopes of Ag_3^+. As the beam current increases, the baseline noise becomes increasingly inflated in a complicated way with a series of local maxima. The pattern is not symmetric with respect to the ion-peak locations and the 100-pA and 200-pA patterns are not "in phase". An expanded view of the mean signal near the ion peaks is presented in **Supplementary Figure 6b** and it exhibits periodicity of approximately

700 points, or equivalently, about 340 Hz if plotted on the frequency scale. The very rapid increase in baseline noise with beam current in this region suggests that attempting to improve sensitivity by increasing the ion count may prove counterproductive.

In addition to the inflated noise magnitude near the Ag_3^+ peaks, the noise becomes correlated with the level of correlation increasing with beam current. A portion of the correlation matrix for 200-pA data binned by a factor of 480 in the spectral domain is displayed in **Supplementary Figure 6c**. The effect is noticeable in the data. **Supplementary Figure 6d** plots the signal for 1000 depths at two representative points in the correlation matrix. The noise at 329.7472 Da exhibits strong positive correlation with the noise at 318.6397 Da but equally strong negative correlation with the noise at 323.6520 Da.

The remaining panel **Supplementary Figure 6e** elaborates on a comment made whilst discussing different data representations. Recall that data can be extracted from the data files as either spectral peak profiles or centroids. In principle, a centroid could be estimated from a peak profile by, for instance, 3-point quadratic interpolation centered on the peak maximum. When this is attempted for present data, a complicated relationship emerges between the extracted and estimated centroids. The mean ratios of extracted to estimated centroid over three 2800-channel spectral windows well separated from ion-induced peaks are plotted for each of the 3 beam currents in **Supplementary Figure 6e**. The extracted centroids are systematically larger than the estimated centroids and the ratios exhibit a periodicity of about 700 channels (like the noise near intense peaks described above). The magnitude and period of the ripples are relatively independent of both mass and beam current.

The final experimental artifact we mention involves slight discontinuities in instrument response with mass. **Supplementary Figure 7a** shows the mean spectrum of the 200-pA data binned by a factor of 60 in the spectral domain in the mass range just below the intense Ag_3^+ peaks. Discontinuities are apparent near 282 Da and 316 Da. The same data normalized by the probability that a centroid is non-zero is plotted in **Supplementary Figure 7b** and accentuates the discontinuity near 282 Da. The magnitude of the discontinuity appears to increase with beam current as it is smaller in the 100-pA data and effectively absent from the 50-pA data. The Orbitrap processing software estimates the noise level for the silver datasets in 50-kHz blocks for purposes of setting the peak-detection threshold. The observed discontinuities fall precisely on the boundaries of one of these blocks. It is likely that the discontinuities are an artifact of the noise estimation algorithm.

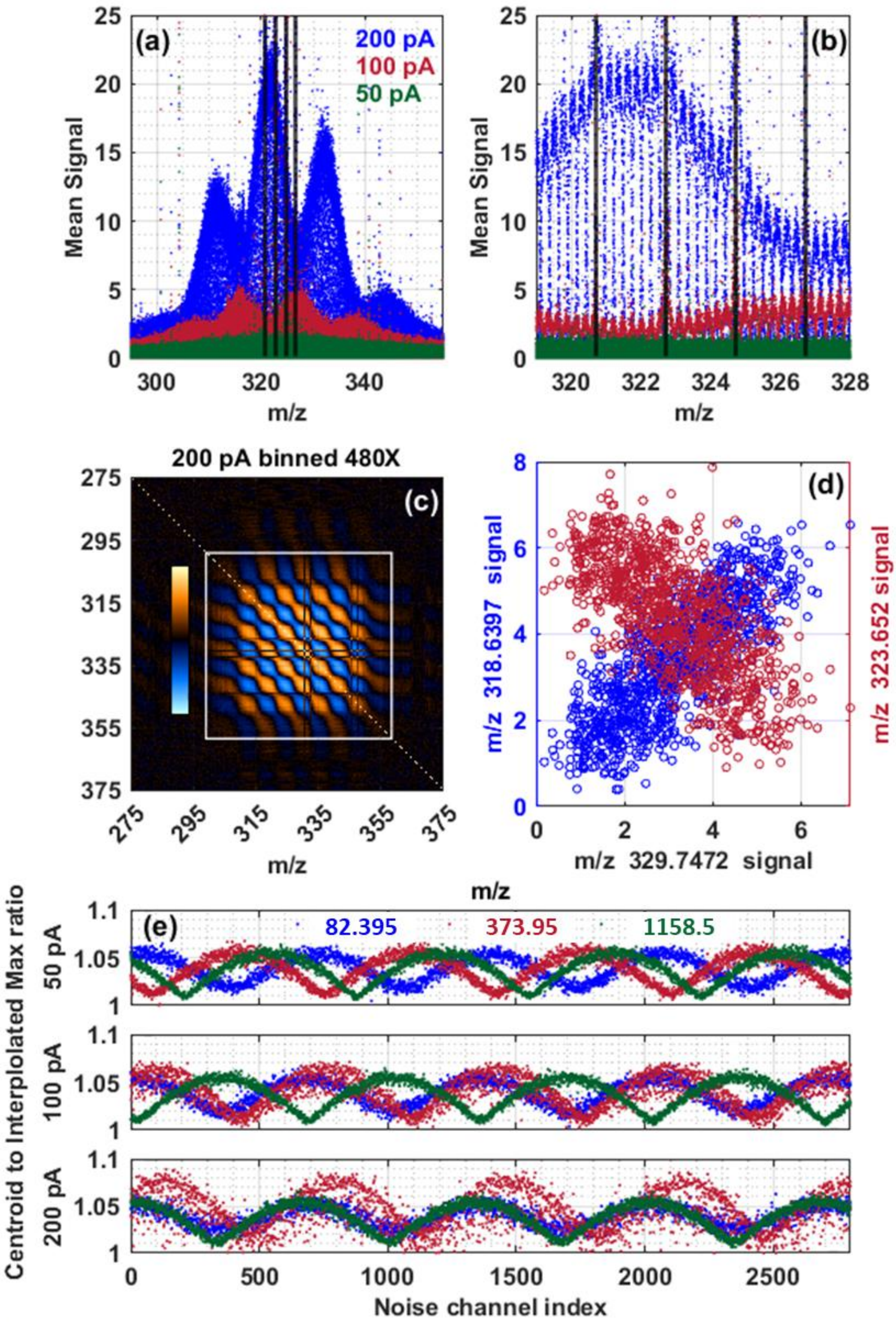

Supplementary Figure 6. (a) Mean signal in the vicinity of the four Ag_3^+ isotope peaks (vertical lines). (b) Expanded view of the mean signal showing an approximately 700-point periodicity. (c) Correlation matrix of the 200-pA data binned by a factor of 480. The region enclosed in the white box contains the mass interval in (a). (d) Representative signal correlations corresponding to + and – correlation regions of (c). (e) Variation in the centroid to interpolated-peak maximum ratio in select 2800-channel noise-only mass intervals. The periodicity is about 700 points.

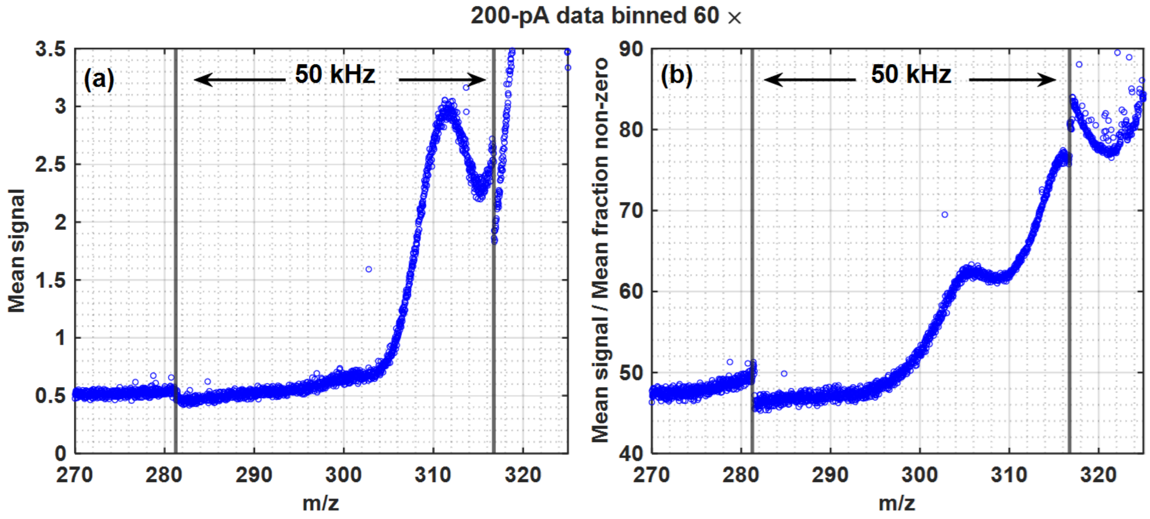

Supplementary Figure 7. (a) Mean spectrum of the 200-pA data binned by a factor of 60 in the spectral dimension. (b) Mean spectrum normalized by the probability that a centroid is non-zero. Both exhibit discontinuities near m/z = 282 and 316 which corresponds to a span of 50-kHz as shown.

Supplementary Note 4: Spectral profiles, centroids and peak lists

OrbiSIMS data can be accessed in several formats. These include full profiles of mass spectral peaks retained in the dataset, peak centroids, and peak lists. The latter integrate peak centroids within predefined mass intervals. For relatively low signal intensities where detector noise is significant, it is important to understand how these types of data differ to avoid pitfalls and properly interpret the results. We will begin by discussing the effects of time-domain apodization and zero-padding on mass spectral peaks in the magnitude spectrum. Next, we consider spectral profiles of noise “peaks” and argue that such data are not representative of the noise distribution, justifying our use of centroid data in the main text. Finally, a few examples where centroided data appear to misrepresent the underlying spectral profiles are presented. These suggest that an external centroiding algorithm applied to the profile data might be advantageous in some cases although we did not pursue that idea here.

The silver depth-profile transients were acquired at 4.096 MHz for 512 ms yielding a time-domain record of length $N = 2^{21}$. To reduce spectral leakage, a Hanning window $w(n)$:

$$w(n) = \sin^2\left(\frac{n}{N}\pi\right) = 0.5 - 0.5\cos\left(\frac{2n}{N}\pi\right), \quad n = 0, 1, 2, \cdots, N-1$$

was applied and the windowed signal was triple-zero-padded to final length $N = 2^{23}$. The Hanning window reduces the noise power by a factor of 3/8 and padding reduces the power

spectral density by an additional factor of four. **Supplementary Figure 8a** shows the effect of windowing and zero-padding independent and identically distributed gaussian noise AWGN in the time domain on the corresponding noise distribution in the magnitude spectrum. Rayleigh-distributed noise with parameter σ in the processed data implies a noise standard deviation of $\sqrt{32/3}\,\sigma$ in the time domain. All statistics reported in the text refer to the processed data. Of greater consequence is the fact that windowing correlates the noise in the frequency domain via convolution with the Fourier transform of the window. **Supplementary Figure 8b** shows how, given a sample of AWGN, the extent of autocorrelation in the magnitude spectrum increases with windowing and padding. The autocorrelation has two effects of interest. First, the FWHM of a noise “peak” is expected to be about eight channels independent of frequency. Second, it becomes very unlikely that two local maxima will be observed within any given 5-channel interval. This exclusion effect is illustrated in **Supplementary Figure 8c**, which compares the autocorrelation of a 2800-channel interval of centroided pure noise in the 50-pA dataset with a simulation of the same. The small sidebands for the measured centroids suggest that we have not fully captured the centroiding process in our simulations.

A small segment of the (uncensored) simulation of the 50-pA noise interval is shown in **Supplementary Figure 8d**. Note that there are large variations in “peak” shape, but that for relatively isolated peaks, the peak widths are reasonably consistent with the expected 8-point FWHM. The corresponding segment of the measured 50-pA data as obtained in the peak-profile representation are displayed in **Supplementary Figure 8**e. Due to thresholding, only the most prominent peaks are captured. The vast majority of peak profiles in the complete dataset are fully defined by eight points of which only five or six are non-zero, notably fewer than the expected FWHM. Some runs of non-zero points are longer; however, they tend to represent overlapped peaks as will be illustrated later. Isolated peaks also appear to have a more uniform shape than would be expected based on the simulation results. Most importantly for our purposes, the peak profiles are not subject to the threshold. This is illustrated in **Supplementary Figure 8f**, which compares a histogram of the 50-pA noise interval in the peak-profile representation with the same data exported as centroids (effectively, the local maxima). Our conclusion is that the peak profiles are not representative of random noise. Rather, they capture several points correlated with each local maximum found in the extreme tail of the noise distribution. As such, the peak-profile data profiles should not be expected to provide reliable information about the noise statistics, and we have used centroided data exclusively in our analysis.

Supplementary Figure 9 overlays centroided mass spectra from all 1000 depths of the 200-pA dataset in the vicinity of the $^{107}Ag_5{}^{109}Ag_5{}^{+}$ peak. The centroids within the 10-channel central peak window represent ion-induced signal plus noise, whereas the remaining channels are just noise. The threshold is plainly evident and applies to all data equally. The noise channels are approximately 99% sparse meaning that pure-noise channels contain about 10 non-zero centroids on average. The absorption mode spectral representation begins when the data exceeds three times the threshold. The magnitude-to-absorption-mode transition is indicated in the figure and suggests that it is highly unlikely that a pure-noise peak will be in absorption mode. In other words, the noise statistics should be representative of the magnitude spectrum. Most of the ion-induced peaks will be in absorption mode, although some fall below the onset level, and, in fact 32 of 1000 total depths register precisely zero signal within the window. The improbability that two peaks will be found in close proximity in a single mass spectrum, as outlined above, is the reason that noise appears absent from the peak window in channels adjacent to the ion peak. There is some jitter in the mass location of the ion peak. To accommodate this variability, peak lists can be defined using peak windows, as shown, with all centroids in the window summed to produce a single peak.

Aside from the general relationships among profiles, centroids and peak lists outlined above, situations can arise where peak heights estimated from the different data representations are significantly discrepant. **Supplementary Figure 10** illustrates several such cases. In (a), two centroids fall within the ion-peak interval. One corresponds to the ion peak and the other corresponds to noise. When exported from the peak list, the peak height is precisely the sum of the two centroids. That is, the peak height overestimates the true ion signal by >25% here. Panel (b) illustrates a related case where two noise centroids fall in a single interval, each corresponding to a local maximum of the profile. As an exported peak, the noise intensity would be about double the intensity of the individual centroids extending the tail of the noise distribution to higher intensities. The last panel (c) contains a noise profile that is unusually asymmetric. It appears to be a single peak with a shoulder but has only a single local maximum and is represented by a single centroid. It is likely that the centroid misrepresents actual noise distribution within the window.

Additional observations with respect to **Supplementary Figure 10** include (1) the ion peaks in (a) and (c) are relatively symmetric but the centroids are noticeably greater than the profile maxima, and (2) the size of the discrepancy seems to vary. This is a direct manifestation of the systematic variation in the centroid-to-interpolated-peak-maximum ratio illustrated in **Supplementary Figure 6e**.

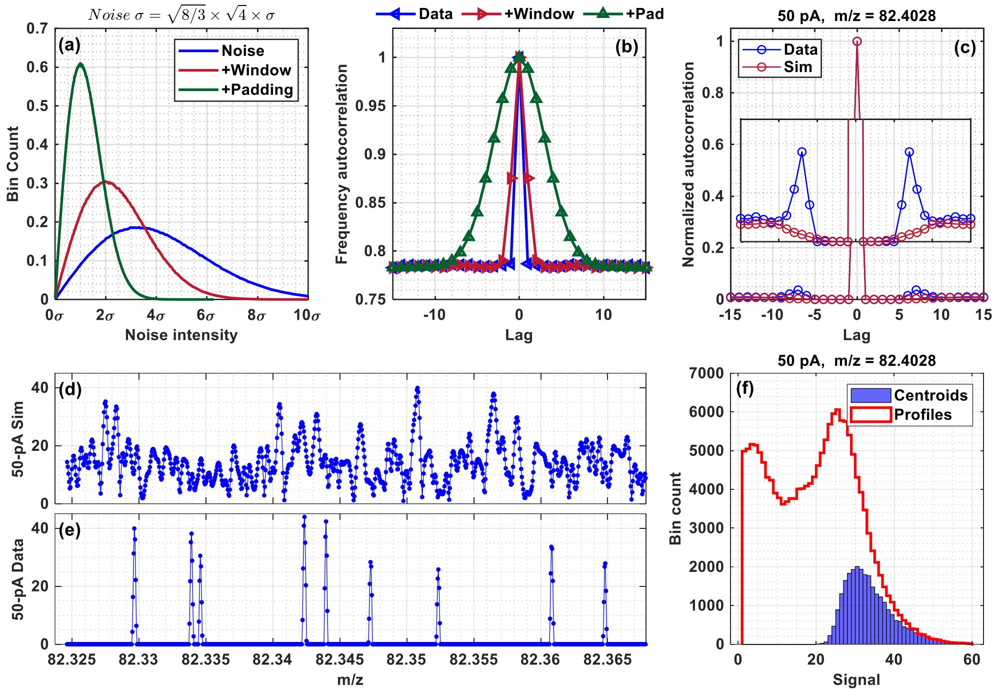

Supplementary Figure 8. (a) Applying the Hanning windows and triple-zero padding the time-domain signal reduces the apparent noise level in the magnitude spectrum. (b) Time-domain windowing correlates the data in the frequency domain, and zero-padding interpolates the magnitude spectrum to improve the characterization of the peaks. The expected FWHM is about 8 channels. (c) Autocorrelation of centroid data in a 2800-channel interval of pure noise. Owing to windowing and zero-padding, it is highly unlikely that two centroids will be observed within a 5-point interval. (d) Simulated noise without thresholding. (e) Representative spectral profiles in the 50-pA dataset. Most profiles are defined by either 5 or 6 non-zero data points. (f) Histograms of the exported profiles and centroided mass spectra from a 2800-channel interval of pure noise in the 50-pA dataset. The profiles do not exhibit a threshold.

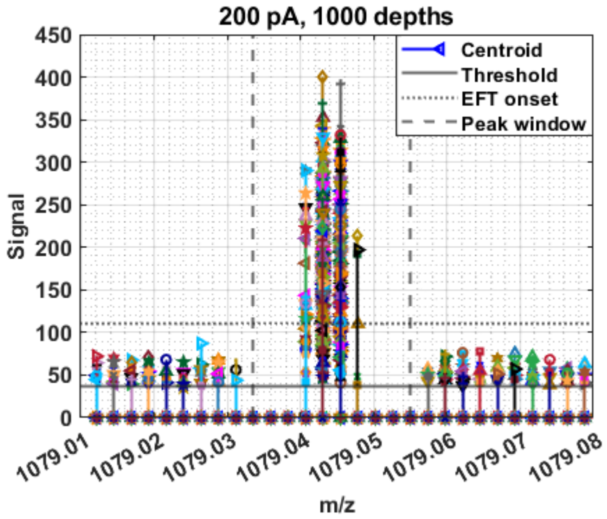

Supplementary Figure 9. Overlay of all centroids from the 1000 depths of the 200-pA dataset in a window surrounding the $^{107}Ag_5{}^{109}Ag_5{}^+$ peak. The solid and dotted lines correspond to the noise threshold and onset of EFT processing, respectively. The vertical dashed lines define a 10-point peak-list interval. All centroids with the interval are summed to produce the peak height.

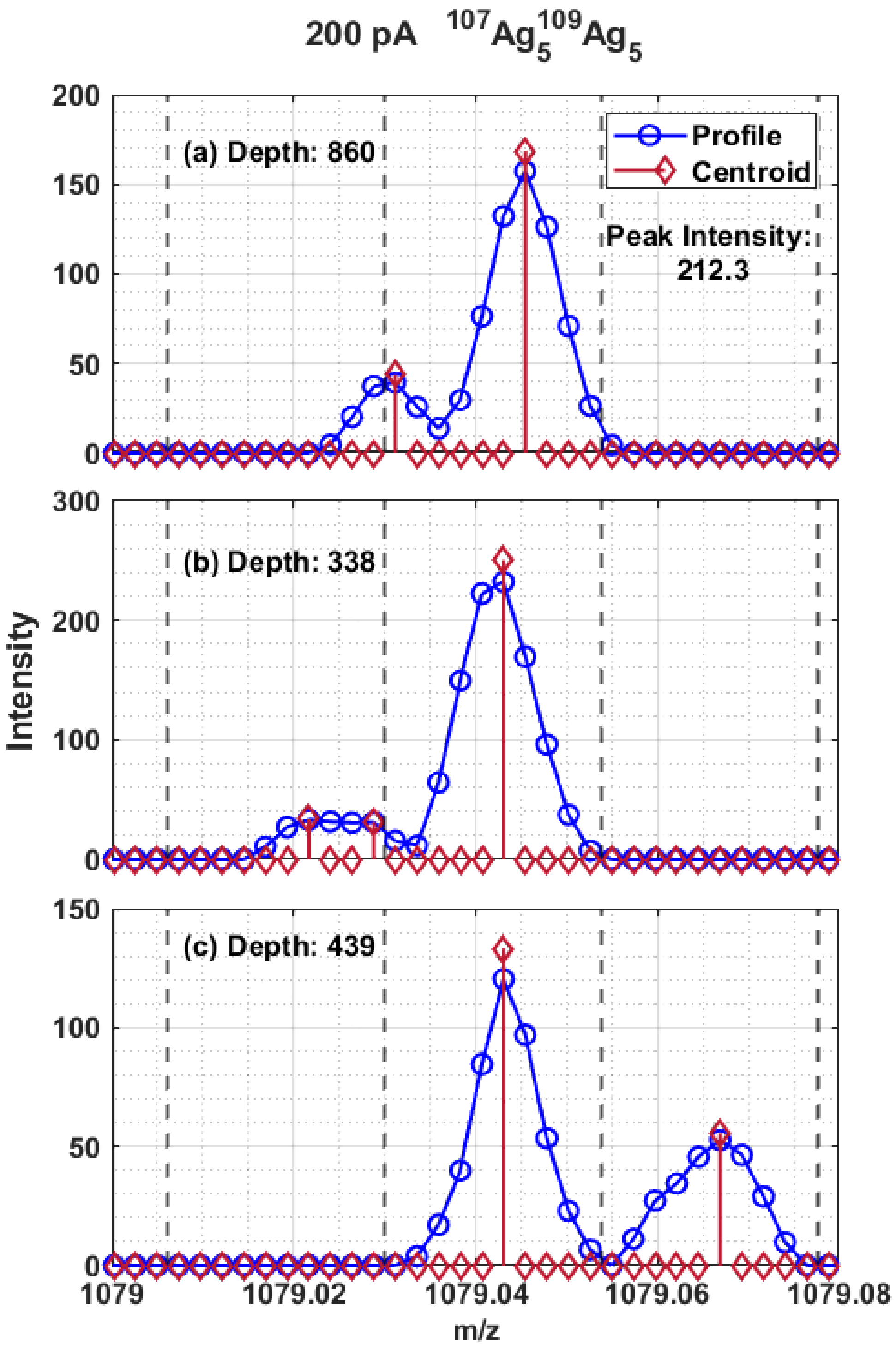

Supplementary Figure 10. Illustration of some discrepancies among signal intensities obtained from peak profiles, centroids, and peak lists for selected mass spectra near $^{107}Ag_5{}^{109}Ag_5{}^{+}$. The vertical dashed lines define 10-point mass intervals over which centroids are summed to produce a peak intensity given a peak list. (a) Two centroids fall within the ion peak interval. The exported peak intensity is the sum of an ion-induced peak and a noise peak. (b) Two centroids fall within a single pure-noise interval approximately doubling the apparent intensity of the noise peak. (c) A noise profile containing a peak within a shoulder is misrepresented by a single centroid.

Supplementary Note 5: Statistics of binned noise

Each mass-spectral peak exported from a peak list sums all centroids contained within the specified mass interval to produce a single intensity value. In the case of random noise, it should be expected that multiple non-zero centroids will be contained within single intervals as the interval width becomes large. That is, the statistical properties of such noise "peaks" will vary with details of the peak list. One consequence of the noise thresholding process is that the probability *p* that a non-zero centroid is detected in any single pure-noise mass channel is small. However, if a non-zero centroid is detected it will add some increment with average value *A*, which is greater than the threshold, to the total signal.

If *n* mass channels are binned, the distribution of the binned signal *X* is expected to follow a scaled version of the binomial distribution with:

$$\mathrm{mean}(X) = Anp$$
$$\mathrm{variance}(X) = A^2np(1-p) = A(1-p)\mathrm{mean}(X)$$

The latter expression indicates that a log-log plot of variance vs mean should be linear with slope 1 and intercept $\log_{10}\big(A(1-p)\big)$. **Supplementary Figure 11a** shows this plot for a 3000-channel noise window of the 50-pA dataset for a variety of binning factors. Here, the slope of the line overlayed on the data has been fixed at unity, and assuming *p* = 0.0128, the observed fraction of non-zero centroids in the window, the additive increment *A* is approximately 39. The expression for variance can also be rearranged to give:

$$\frac{\mathrm{variance}(X)}{\mathrm{mean}(X)^2} = \frac{1-p}{p} \cdot \frac{1}{n}$$

As shown in **Supplementary Figure 11b**, a plot of the left-hand-side vs the inverse binning factor is linear. The line is a prediction of the slope based on the observed fraction of non-zero centroids and the agreement with the data is good. An estimate of *p* from the slope of a fitted line differs by about 1% from the directly observed value.

As detection limits are frequently defined in terms of the noise standard deviation, the statistical model for binned noise is useful for assessing the impact of peak-interval size on detection sensitivity. This is likely most important in imaging applications where a major chemical component may not be present at all locations. In other words, mass spectral peaks that must be captured in the analysis may, nevertheless, contain many pixels that are purely noise, having no signal induced by the corresponding ions. **Supplementary Figure 12**

provides an example suggesting that care should be taken when selecting the peak-list intervals for a given application and interpreting low-intensity mass peaks.

The relationship between the mean and variance of the noise is fundamental to the analyses described in this article. **Supplementary Figure 12a** shows how the window means and variances of non-zero noise elements increase with the width of the peak interval. For intervals ranging up to 6 or 7 bins, independent of mass, the ratio of variance to mean is approximately constant. In this regime, it is highly unlikely that more than a single noise centroid will be present in any given interval. Beyond this point, it becomes more likely to have multiple non-zero centroids in a single interval and the variance becomes greatly inflated with respect to the mean. For variance-based multivariate statistical methods such as PCA, increasing the bin width may lead to a several-fold decrease in detection sensitivity.

A second, more subtle, problem with large interval widths is illustrated in **Supplementary Figure 12b**. One common approach to exploring data is examining its histogram. In an image, for instance, a multi-modal histogram of a particular ion suggests the presence of multiple classes of pixels containing that ion. As shown in the figure, the histogram of binned pure noise can also be multimodal. It is tempting to interpret this histogram as mostly noise with a small amount of a second component added in. That would be incorrect, however. Here, a very large zero peak (not shown) represents those intervals that contain no non-zero centroids. The major peak with mode near 35 contains those intervals with a single centroid and the minor peak near 75 largely consists of intervals containing two centroids. In other words, there are multiple classes of data elements, but they are defined via the thresholding and binning processes rather than anything of chemical significance.

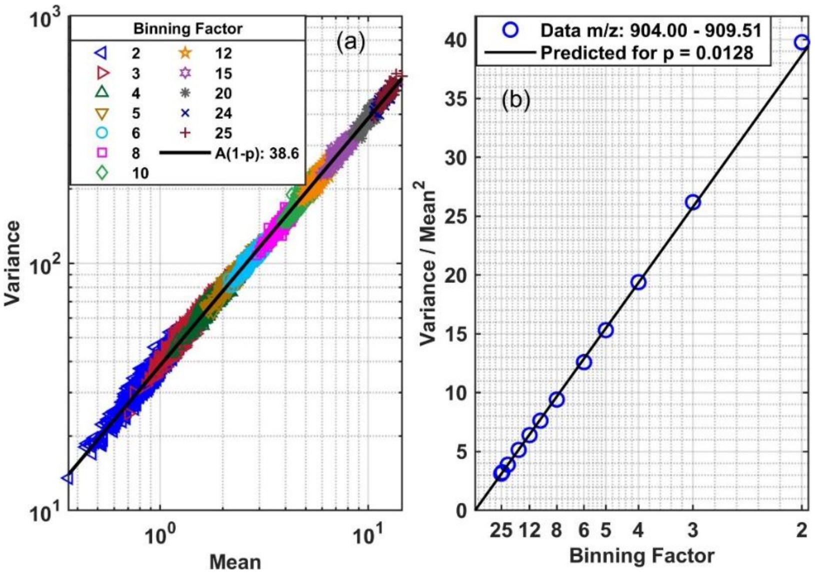

Supplementary Figure 11. (a) Variance vs. Mean relationship for a 3000-channel noise window in the 50-pA dataset as it is binned to different extents. The intercept of the line is related to the average increment that a non-zero centroid adds to the data. (b) The ratio of the variance to the squared mean has a linear relationship with the inverse binning factor. The line is a prediction based on the observed fraction, 0.0128, of centroids that are non-zero in this window.

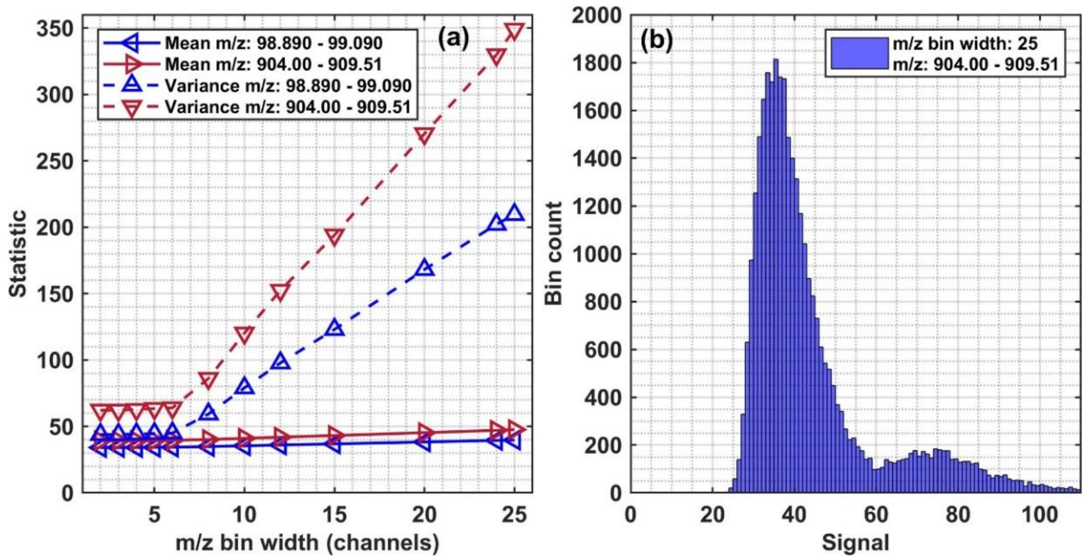

Supplementary Figure 12. (a) Mean and variance relationships of non-zero centroids in two 3000-channel noise windows of the 50-pA dataset as the windows are binned to varying extents. (b) Histogram of non-zero centroids in one of the windows binned by a factor of 25. The major peak corresponds to binned data elements with contributions from a single centroid. Data elements in the smaller peak sum 2 or more centroids.

Supplementary Note 6: Simulation of the silver profiles and thresholding

A simulation based on the probabilistic model was constructed to compare the model with the measured data. As detailed in the main text, 34 distinct ion clusters with a total of 192 masses were captured in the mass spectral dataset. Simulation “truth” was established by matching the predominant peak in each cluster with the observed mean spectrum and calculating the intensities of the remaining isotopic combinations using the natural abundances of Ag. Time-domain signals were constructed by summing abundance-weighted, zero-phase pure sinusoids and adding random noise. The mass range from 80 to 1200 Da was simulated with the corresponding frequency scale defined by fixing the Nyquist frequency to correspond to approximately 50 Da. Each simulation required four parameters relating signal to noise: the ion-to-signal scaling factor *A*, the excess variance due to an overdispersed total number of ions σ_N^2, and the white and 1/f components of detector-related variance σ_W^2 and σ_F^2, respectively. The simulation is run at sampling rate of 4.096 MHz for 0.512 s, and the major steps include:

1. Draw the total number of ions from a negative binomial distribution with the desired mean and variance.
2. Partition the ions into mass bins using a multinomial random number generator.
3. Scale the number of ions to a signal amplitude and generate noise-free time-domain signals.
4. Add noise. The noise is generated from uniform gaussian random numbers by shaping its power spectrum to have the desired properties and obtaining noise samples by inverse FFT.
5. Apply the Hanning window and triple zero-pad the time domain signal.
6. Perform an FFT
7. Calculate the single-sided magnitude spectrum.
8. Extract peak centroids from the magnitude spectrum.
9. Sum centroids within 5-point mass intervals to mimic an exported peak list.

The centroiding algorithm used in step 8 itself entails several sub-steps:

8a. Partition the frequency range into intervals over which the noise in the magnitude spectrum is assumed constant and Rayleigh-distributed. Here the intervals are taken to be 50 kHz wide to match the apparent interval size used by the Orbitrap processing software for the silver data.

8b. In each interval, estimate the Rayleigh parameter σ, which is equal to the time-domain noise standard deviation. The simulation uses the maximum likelihood estimator MLE for σ^2, which is one-half the mean of the squared data.

8c. Set a peak-detection threshold. The simulation threshold was taken to be 2.54σ to approximately match the sparsity of the simulation with that of the data.

8d. Peaks are detected as sequences of three points centered on local maxima such that all three points exceed the threshold.

8e. The centroid intensity is obtained via 3-point quadratic interpolation of the peak values.

Obtaining a good estimate of the detector-noise standard deviation in real-time as data is acquired poses significant challenges. Owing to 1/f noise, σ is not truly mass-independent within an interval and, in addition to noise, intervals may contain any number of ion-induced peaks with widely varying intensities. The estimation approach used in the simulation was inspired by the patent literature.[18] An initial estimate of σ is obtained from the highest frequency block and a peak-detection threshold set. Then, starting with the highest frequency block, data exceeding the threshold are assumed to be ion-induced and are discarded with σ for the block updated to the MLE of the retained data. The threshold is reset based on the updated σ and is used to initialize the estimate for the subsequent frequency block. An important consequence of this block-wise noise estimation procedure is that it has memory. In other words, the estimate of σ obtained in any given block is influenced to some extent by the estimates made in prior blocks.

Supplementary Figure 13a compares the fraction of non-zero centroids in the 50-pA dataset with a simulation of the same. The zigzag appearance has a 50 kHz period and is a consequence of assuming the noise level is constant within each interval. At high frequency (low mass) the data and simulation agree reasonably well. Given that σ is accurately estimated, our model predicts the fraction of centroids exceeding the threshold should, on average, be constant. At low frequencies (high mass), the data and simulation begin to diverge in a way suggesting that the algorithm the Orbitrap MS uses is underestimating the noise level. Other discrepancies between the simulation and data are also seen at the higher beam currents. **Supplementary Figure 13b** compares the 200-pA data and simulation with the abscissa having a mass scale. In this case, it appears that the Orbitrap MS is overestimating the noise level in mass intervals on the high side of the very intense Ag_3^+ peaks. This is likely a consequence of the memory effect noted above. In general, we expect the threshold to increase with mass and this is the case for the 200-pA data as shown in **Supplementary Figure 13c**. The thresholds are taken to be the point at which the rising edge of the histogram exceeds 70% of its maximum value. Thresholds estimated in this manner are

displayed in **Supplementary Figure 13d** for the 200-pA data binned into intervals 1440 frequency channels wide. It should be noted that some noise information is stored in the Orbitrap RAW file. Several values extracted from the 200-pA RAW file are overlaid on the thresholds in **Supplementary Figure 13d**. The two plots generally coincide suggesting that the stored information represents the instrument-estimated thresholds.

Supplementary Figure 14a-c compares the observed and simulated variance vs mean curves for the three different beam currents. The agreement is excellent. One seeming outlier in the 100-pA data corresponds to Cs+ is discussed at length in **Supplementary Note 7**: Description of data scaling and transformations for multivariate analysis and comparison using silver datasets. The distributions of the data at low signal intensity are compared in **Supplementary Figure 14d-f**. The simulation reproduces the proper thresholds and general shapes of the low-signal histograms. As eFT is not included in the simulation, the notch present in the measured data at three times the threshold is not mirrored in the simulation. According to the probabilistic model, overdispersion is caused by all ions varying in tandem and results in the data being correlated. Simulated and data correlation matrices are compared in **Supplementary Figure 14g-i**. All diagonal elements in the images are unity. Again, the agreement is excellent. The simulation clearly provides a faithful representation of the overall data distribution in this regime. The simulation is self-consistent in the sense that analysing the simulated data using the methods outlined in this paper recapitulates the input parameters to within a few percent. However, the simulation does not include the observed instrument-induced anticorrelation of the small and large clusters, thus data deflated by the principal component describing the anticorrelation was used for this comparison.

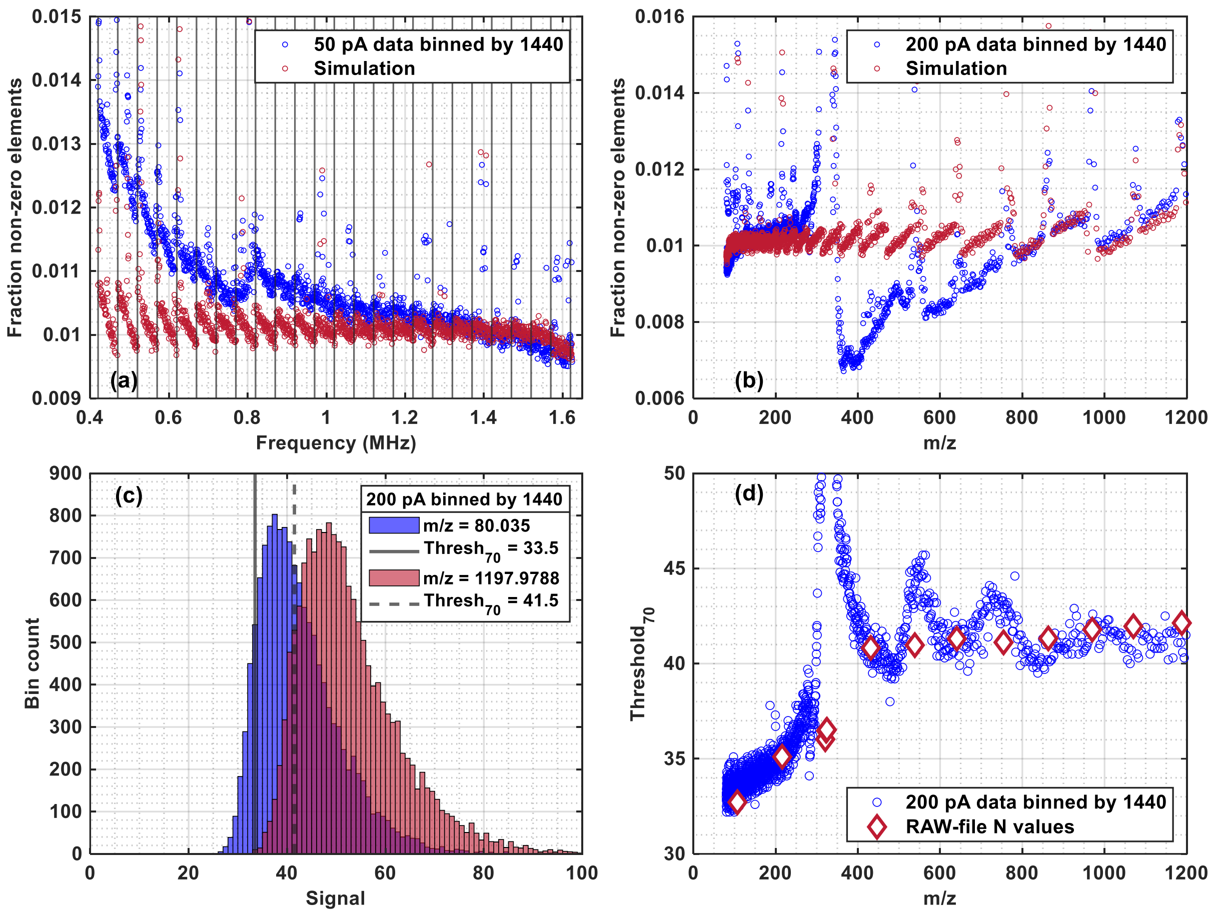

Supplementary Figure 13. (a) Fraction of non-zero centroids for the 50-pA data set compared with a simulation. The vertical lines define 50 kHz intervals over which the noise level is estimated. (b) Similar plot for the 200-pA dataset but presented as a function of mass rather than frequency. (c) Histograms of centroid intensity in two intervals devoid of ion peaks at opposite ends of the mass range. The vertical lines are thresholds defined as the point at which the rising edge of the histogram exceeds 70% of its maximum. (d) 70% thresholds for all binned intervals of the 200-pA dataset. The overlaid diamonds are N-values obtained from the Orbitrap RAW file.

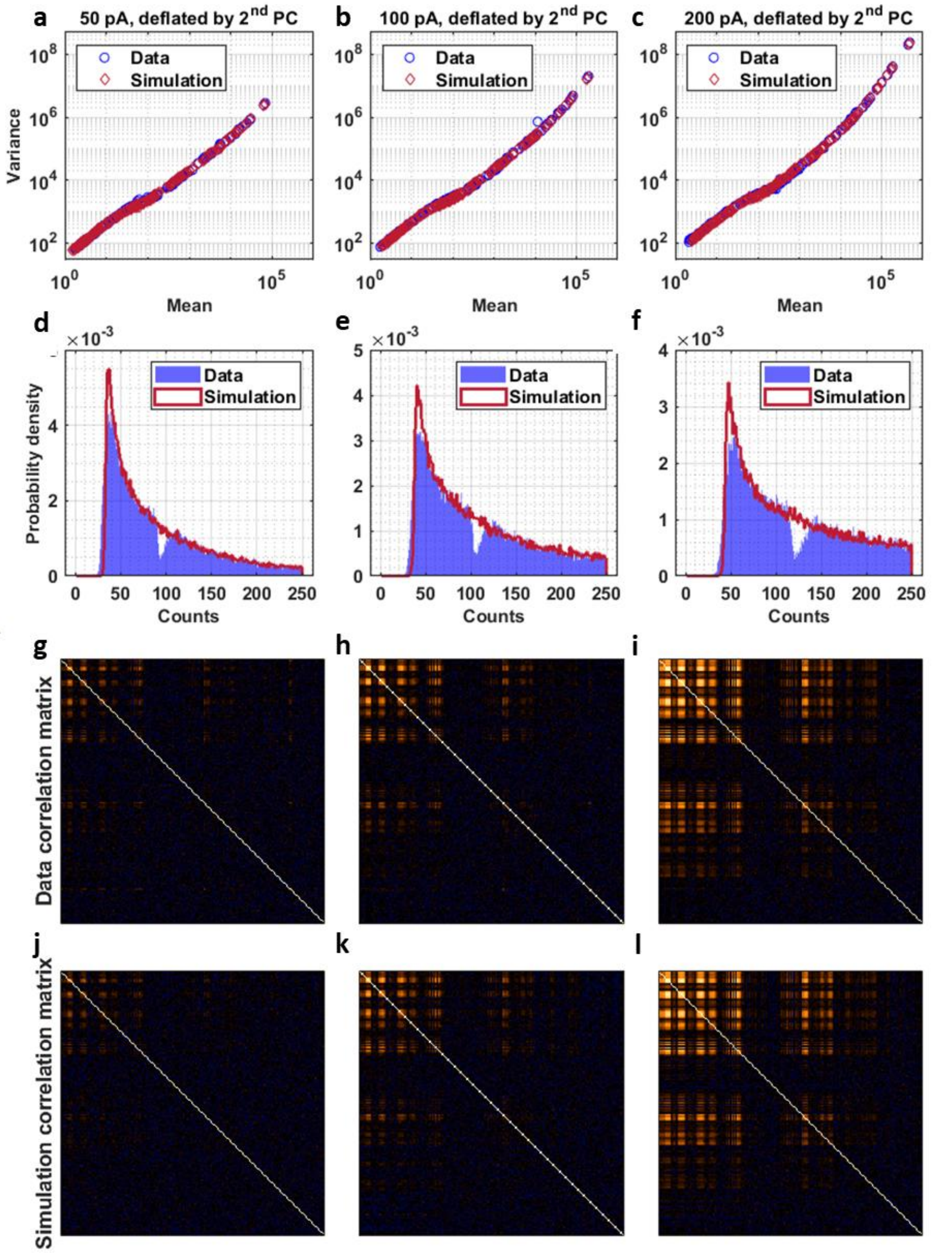

Supplementary Figure 14: Comparison between observed and simulated data: **a-c** Variance vs mean curves for the three different beam currents. **d-f** Distributions of the data at low signal intensity. **g-l** Correlation matrices

Supplementary Note 7: Description of data scaling and transformations for multivariate analysis and comparison using silver datasets

Recall the model of a mass-spectral dataset presented in Eq. (25). Subtracting the mean from both sides, taking cross-products assuming the signal and noise are not mutually correlated and dividing by the number of spectra *m*:

$$\frac{1}{m}(\mathbf{S}-\mathbf{1}_m\boldsymbol{\mu}^T)^T(\mathbf{S}-\mathbf{1}_m\boldsymbol{\mu}^T)=\frac{1}{m}\mathbf{W}\mathbf{Z}^T\mathbf{Z}\mathbf{W}^T+\frac{1}{m}\mathbf{S}_E^T\mathbf{S}_E$$
$$\mathbf{C}=\mathbf{C}_0+\mathbf{C}_E \qquad (38)$$

C and $\mathbf{C}_E$ are the sample and noise covariance matrices, and $\mathbf{C}_0$ is the covariance of the signal, the deterministic part of the data. PCA performs an eigenanalysis of **C** and orders the principal components by eigenvalues, the amount of variance that each component captures. PCA makes no distinction between variance arising from signal and that due to noise. Thus, chemically informative components can be interspersed among components describing only noise making it difficult to distinguish noise from minor systematic variations. This point can be illustrated with the silver-depth-profile data. **a** compares the eigenvalues of the 50-pA dataset with those of the model noise covariance matrix given in Eq. (21). Aside from the leading eigenvalue, which accounts for variance due to the mean signal, the eigenvalues largely coincide in the high-signal regime where the noise model is valid. Typically, the number of “significant” components is estimated from the break in the eigenvalue curve. We would likely choose a single component in the present case. **b** displays the three leading principal-component loadings. The first corresponds to the mean spectrum, as expected, and the second is clearly noise, namely, anticorrelation of the two main isotopes of Ag_3^+, the cluster with the highest signal level. The third, however, relates to the real instrument-induced variation reported earlier for all three datasets.

A similar but composition-related example is provided by the 100-pA dataset. Cesium surface contamination is present. However, unlike the silver-containing clusters that show no systematic intensity variation with depth, Cs^+ intensity decreases with depth as shown in **c**. Using standard PCA, the variance of the average Cs^+ intensity is captured by the mean-spectrum component but the idiosyncratic depth-dependent contribution to the variance is not discovered until the 11th most significant PC as shown in comparison with the centered Cs^+ depth profile in **c**. From an analyst’s point of view, sorting through all principal components to find interesting features would be no different than sorting through all

individual ion depth profiles. In fact, it would likely be more difficult owing to the abstract character of many components induced by the intrinsic PCA orthogonality constraints.

The fundamental problem in the foregoing example is that high-intensity peaks are given too much influence in the analysis owing to large variances reflecting counting noise. As noted earlier, PCA makes the implicit assumption that data are homoscedastic, that is, the noise covariance matrix is proportional to the identity matrix. This assumption is severely violated in count-based measurements. The strategy to make more effective use of PCA is to preprocess the data with either transformations or weighting to make the noise appear uniform, independent of signal intensity. For low-intensity signals in ToF-SIMS that are Poisson-distributed, a scaling procedure[19] has been successfully employed. At higher signal levels where detector dead-time effects become important, an alternative method that involves both data transformation and scaling has been provided.[20] Our goal is to devise and evaluate similar procedures in the context of OrbiSIMS data.

Returning to the covariance decomposition of Eq. (38), both sides of the equation can be right-multiplied by the inverse of the error covariance matrix to give:

$$\mathbf{C}\mathbf{C}_E^{-1} = \mathbf{C}_0\mathbf{C}_E^{-1} + \mathbf{I} \tag{39}$$

The quantity $\mathbf{C}_0\mathbf{C}_E^{-1}$ is a matrix version of the squared signal-to-noise ratio. Thus, eigenanalysis of the sample covariance matrix weighted in this fashion should order components by S/N rather than by variance, increasing the probability that interesting components will be shuffled to the front. Eq. (39) presents a non-symmetric eigenvalue problem. An equivalent symmetric eigenvalue problem is obtained by both left and right multiplying Eq. (38) by the inverse matrix square root of the error covariance matrix:

$$\mathbf{C}_E^{-1/2}\mathbf{C}\mathbf{C}_E^{-1/2} = \frac{1}{m}\left(\mathbf{C}_E^{-1/2}\mathbf{W}\right)\mathbf{Z}^T\mathbf{Z}\left(\mathbf{W}^T\mathbf{C}_E^{-1/2}\right) + \mathbf{I} \tag{40}$$

The equation is written this way to suggest that eigenanalysis of the cross-product of data weighted by $\mathbf{C}_E^{-1/2}$can be expressed in terms of weighted spectral components. The components in physical space can be recovered *via* the inverse weighting. The remaining question is how to estimate $\mathbf{C}_E$ or its inverse. Three basic approaches to estimating the error covariance matrix are available. These include estimating it empirically from the data itself, using *a priori* knowledge of the noise structure, or employing heuristic methods based on summary statistics of the data.

Machine learning-based covariance estimation

Probabilistic Factor Analysis PFA was described in **Supplementary Note 1**: Expanded description of the data distribution for OrbiSIMS mass spectrometry**,** and it obtains $\mathbf{C}_E$ as a by-product of the matrix factorization algorithm. PFA assumes $\mathbf{C}_E$ is a diagonal matrix and uses a cyclic iterative algorithm to alternately estimate **Z**, **W** and $\mathbf{C}_E$ one at a time keeping the other two quantities constant. Whilst not explicitly a scaling method as described above, the data and **W** are scaled internally as in Eq. (40) during estimation of **Z**. PFA has the advantage that it estimates the uncorrelated part of the variance over the entire intensity range of the silver depth profile, including data that are censored. One drawback of PFA is that empirical estimation of $\mathbf{C}_E$ is a data-intensive enterprise. Many spectra are required to generate good estimates. A more subtle issue is that $\mathbf{C}_E$ is not strictly error *per se*. The diagonal elements of $\mathbf{C}_E$ estimated by PFA are the variances unique to each mass peak. For mass spectral applications with fragments or isotopic variants that are correlated, this is not a problem. However, systematic variation of a unique species that varies independently of everything else in the data will be classified as noise. This is the case with Cs^+ in the silver depth profiles. Having only a single isotope and being the only mass peak that varies with depth, the depth-dependent contribution of the Cs^+ variance adds to the noise estimate. **d** shows that Cs^+ appears to be an outlier in the variance-to-mean plot due to this effect.

A practical disadvantage of PFA is that the number of factors must be specified in advance. This contrasts with PCA, which factors the full data matrix. If the requested number of factors exceeds the number of signal-bearing components, the PFA iteration converges either very slowly or not at all. One way to overcome this difficulty is to use a hybrid algorithm where the inverse square root of the PFA-estimated error covariance is used to scale the data prior to performing PCA. It is expected that $\mathbf{C}_E$ will converge more quickly than the factor matrices and be less sensitive to the rank of the PFA factor model.

PFA assumes the noise covariance matrix is a diagonal matrix. It should be noted that there are other empirical methods for estimating the covariance matrix that are not similarly constrained. Two approaches that have been taken with ToF-SIMS data include the shift-difference procedure inherent to Maximum Autocorrelation Factors MAF[21,22] and the so-called Haar method.[23] Both methods assume the composition is locally constant so any nearest-neighbor intensity differences can be attributed solely to noise. A complete discussion of these techniques is beyond the scope of the paper. However, they can be used effectively where noise is correlated beyond that induced by overdispersion. The main drawback is again the need for large quantities of data to produce good estimates,

particularly as additional numbers, the off-diagonal elements, must be estimated to construct the full covariance matrix.

Model-based noise covariance estimation

Before considering the full probabilistic model derived in this paper, it is instructive to examine the simpler model with error covariance given by Eq. (21), which is suitable for moderate to high signal levels. Here, the noise covariance matrix is a symmetric rank-1 modification of a diagonal matrix. Weighting involves the inverse of the matrix, which can be computed[24], and after some algebra:

$$\mathbf{C}_E^{-1} = \mathrm{diag}(A\,E[\mathbf{s}])^{-1} - \frac{1}{A^2}\left(\frac{1}{R_N^2} + \frac{\mathbf{1}^T E[\mathbf{s}]}{A}\right)^{-1} \mathbf{1}\mathbf{1}^T \qquad (41)$$

This matrix is a rank-1 modification of the diagonal matrix whose elements are the inverse of the scaled mean spectrum. In the limit that R_N the relative standard deviation due to overdispersion goes to zero (*i.e.*, the data are Poisson), only this diagonal term survives. Since the effect of scaling is invariant to a constant scalar factor[19], the appropriate scaling is simply the root-mean scaling approach used in ToF-SIMS, where each spectrum is scaled by the inverse square root of the mean spectrum. Considering the off-diagonal elements, the right-hand term within the parentheses is the total number of ions in the trap and for moderate overdispersion both terms are large numbers of which we take the inverse sum. In the case of the silver depth profiles, the off-diagonal elements are effectively zero and, again, root-mean scaling is appropriate. **Supplementary Figure 15e** shows that the Cs^+ depth variation is captured in the 3rd most significant principal component, and the corresponding loading, which isolates the Cs^+ spectrum, is displayed in **Supplementary Figure 15f**. Root-mean scaling has enabled the three known sources of systematic variation to be captured in the three leading principal components.

Unlike PFA, the model of Eq. (41) is not applicable to the entire intensity range. However, consideration of that model provides the important insight that overdispersion can be safely neglected when deriving a scaling matrix. Recall that the full probabilistic model predicts the measured signal is distributed as a weighted sum of Ricians, and that the variance can be computed according to Eq. (9) in **Supplementary Note 1**: Expanded description of the data distribution for OrbiSIMS mass spectrometry. In the absence of overdispersion, the appropriate weights are obtained assuming Poisson-distributed N. The WSoR-computed variances are given by the curves in **Supplementary Figure 2d-f**. The diagonal of a suitable full-range error covariance matrix can be constructed by interpolating these curves at the

measured peak means. Using this procedure, the three known sources of variation are captured in the three leading principal components at each of the three beam currents.

Heuristic scaling and transformations

Besides the empirical data-driven and model-based methods for estimating the error covariance presented above, several scaling procedures and data transformations have found use in mass spectral imaging and metabolomics work.[25] These include variance and Pareto scaling, and logarithmic data transforms as described in the main text. Generally, these are presented without theoretical justification and are simply employed to achieve a desired effect. We will consider these in the context of the probabilistic model below. In addition, we will briefly discuss the square root transformation, which would seem to be an appropriate choice for count data.

Heuristic scaling methods take the diagonal error covariance matrix to be a peak-wise summary statistic of the full data matrix. Root-mean scaling, which was treated earlier, uses the mean spectrum. Variance scaling and Pareto scaling assume the noise variance is equal to the sample variance and sample standard deviation (square root of the variance), respectively. Unlike the model-based methods, these methods make no distinction between variance arising from the systematic structure of the data and noise. To facilitate the discussion, we will consider the simplified noise variance model of Eq. (41) that neglects detector noise and thresholding. Equation (38) equates the sample covariance matrix to the sum of the signal and error covariance matrices, with the resulting sample variance predicted to be:

$$\mathrm{diag}(\mathbf{C}_S) = \mathrm{diag}(\mathbf{C}_0) + AE[\mathbf{s}]\left(1 + \frac{R_N^2}{A}E[\mathbf{s}]\right)$$

Recall that the goal of scaling is to equalize the uncorrelated part of the noise variance, which is simply $AE[\mathbf{s}]$ Clearly, root-mean scaling, which divides the variance by $E[\mathbf{s}]$ accomplishes this. Dividing $AE[\mathbf{s}]$ by the sample variance and sample standard deviation yields predictions for the noise variance of variance-scaled and Pareto-scaled data, respectively:

$$\mathbf{C}_{\boldsymbol{E}}(S_{variance}) = \frac{AE[\mathbf{s}]}{\operatorname{diag}(\mathbf{C}_0) + AE[\mathbf{s}]\left(1 + \frac{R_N^2}{A}E[\mathbf{s}]\right)}$$

$$\mathbf{C}_{\boldsymbol{E}}(S_{Pareto}) = \frac{AE[\mathbf{s}]}{\sqrt{\operatorname{diag}(\mathbf{C}_0) + AE[\mathbf{s}]\left(1 + \frac{R_N^2}{A}E[\mathbf{s}]\right)}} \tag{42}$$

$\mathbf{C}_0$ is negligible for the silver depth profiles. Then, in the absence of overdispersion variance scaling produces an identity noise covariance, whereas the variance of Pareto-scaled data is proportional to the square root of the mean spectrum. On the other hand, if overdispersion is the largest contributor to the variance, Pareto scaling equalizes the noise variance, and the variance of variance-scaled data is inversely proportional to the mean spectrum. In other words, the relative performances of variance and Pareto scaling will depend on details of the particular data being analyzed. This will be especially true for images where spatial variations captured in $\mathbf{C}_0$ contribute to the sample variance. In general, the justification for Pareto scaling appears to be based on the heuristic argument that it provides results intermediate between two things assumed to be suboptimal: no scaling and variance scaling.[26] Our analysis in Eq. (42) suggests that Pareto scaling is indeed intermediate considering the noise variance of unscaled data is directly proportional to the mean spectrum.

Again, root-mean scaling appears to be the proper scaling method at moderate to high signal levels where detector noise can be neglected. To validate this conclusion, subsets of the data were constructed by removing all mass peaks that contained one or more zeros within the 1000 depths of the profile. The smallest mean signals in the subsets were approximately 250. Root-mean scaling enables the Cs^+ depth variation to be discovered within the first three principal components at all three beam currents. For the silver depth profiles, variance scaling performs similarly suggesting that the signals verge upon the low-overdispersion regime. Here, Pareto-scaled data should have noise variance proportional to the square root of the mean spectrum. Pareto scaling performed best on the 100 pA dataset where the Cs^+ trend is discovered in the 4th PC. The corresponding loading is shown in **f**. The Cs^+ trend was found in the 8th and 7th PC of the 50-pA and 200-pA datasets, respectively. Pareto scaling exhibited very uneven behavior when applied to these simple datasets.

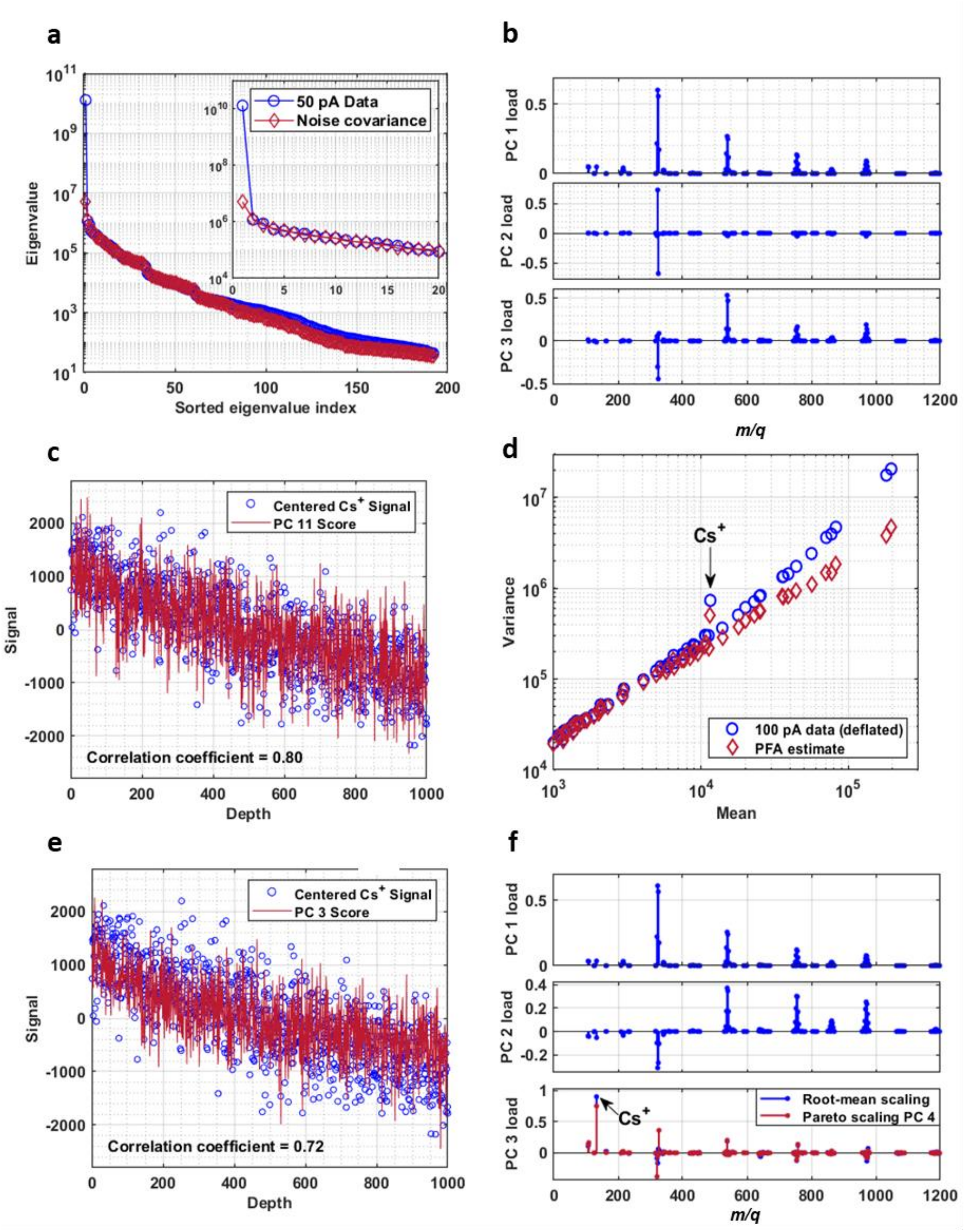

Supplementary Figure 15: a Comparison PCA eigenvalues of the 50-pA dataset against those of the model noise covariance matrix given in Eq. (21). **b** Loadings of PC 1-3 of the unscaled 50-pA dataset. **c** Scores of PC 11 of the unscaled 50-pA dataset compared against centered signal of Cs^+. **d** Variance vs mean plots for the deflated 100-pA data and the PFA estimate, showing the Cs+ is an outlier. **e** Scores of PC 3 of the root-mean scaled 100-pA dataset compared against centered signal of Cs+. **f** Loadings of PC 1-3 of the root-mean scaled 100-pA dataset.

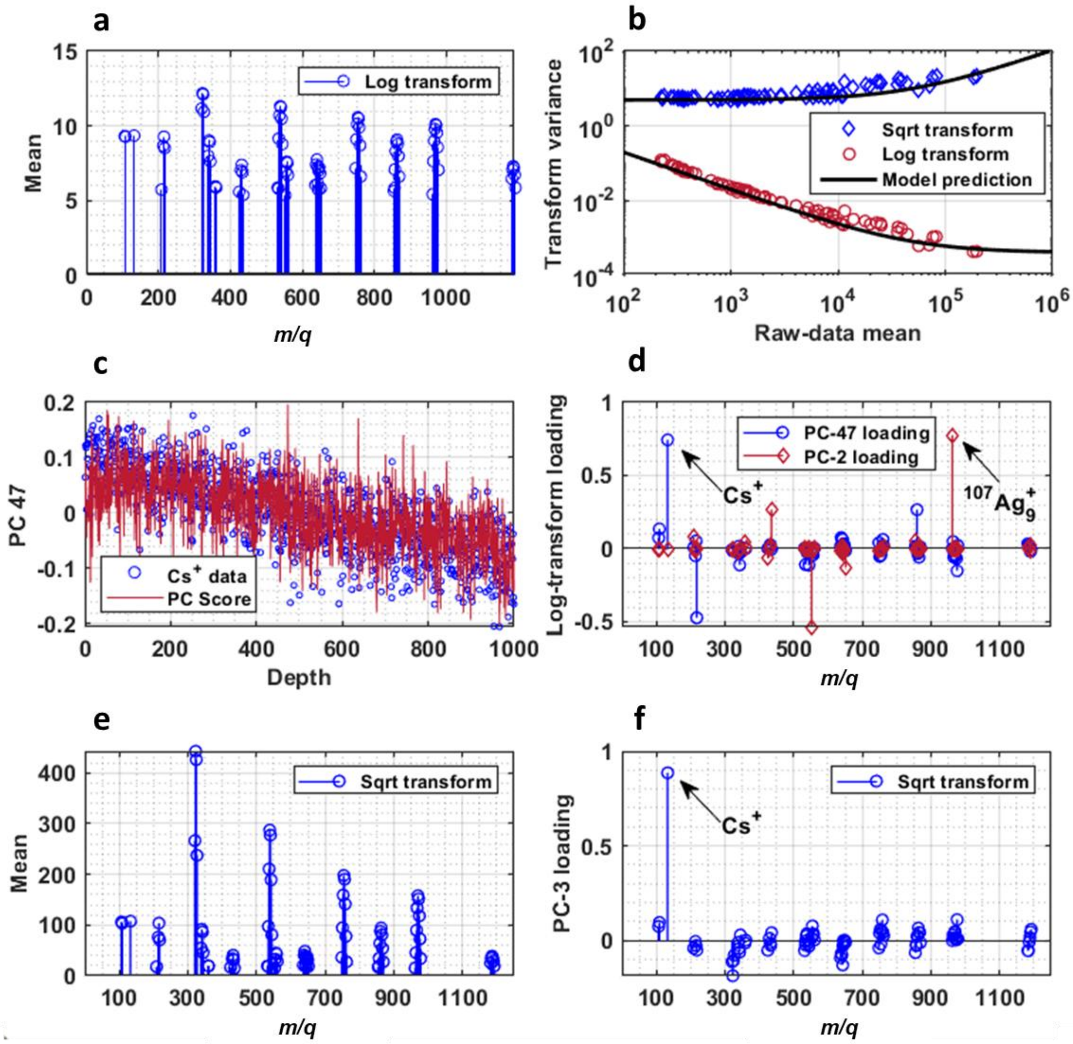

Supplementary Figure 16: a Mean spectrum of the log-transformed data of a 77-peak subset of the 100-pA data (no zeros included). **b** Variance of log and square-root transformed data as function of mean of raw 100-pA data. **c** Scores of PC-47 of the log-transformed data overlaid on the Cs^+ profile. **d** Loadings of PC-47 and PC-2 for the log-transformed data. **e** Mean spectrum of the square-root-transformed data of a 77-peak subset of the 100-pA data (no zeros included). **f** Loadings of PC-3 for the square-root-transformed data.

Taking the logarithm of the data is another approach often used to reduce the influence of large mass peaks. Setting aside the problem of zero-intensity elements in the data matrix, we used the 77-peak uncensored subset of the 100-pA dataset. **Supplementary Figure 16a** shows the mean spectrum of the log-transformed data. The peak intensities appear more equal, as intended, although the monotone nature of the transform ensures the largest peaks remain the largest when transformed. The variance of the transformed data, on the other hand, is not equalized. As shown in **Supplementary Figure 16b**, the variance decreases by over two orders of magnitude from the smallest peaks to the largest. Given the variance model in Eq. (21), the expected value and variance of log-transformed OrbiSIMS data can be approximated using a first order Taylor series expansion:

$$E[\log(\mathbf{s})] \approx \log(E[\mathbf{s}]) - \frac{\mathrm{Var}(\mathbf{s})}{2E[\mathbf{s}]^2} = \log(E[\mathbf{s}]) - \frac{1}{2}\left(\frac{A}{E[\mathbf{s}]} + R_N^2\right)$$
$$\mathrm{Var}(\log(\mathbf{s})) \approx \frac{\mathrm{Var}(\mathbf{s})}{E[\mathbf{s}]^2} = \frac{A}{E[\mathbf{s}]} + R_N^2 \quad (43)$$

The predicted variance agrees well with the observed variance as shown in the figure.

Equation (43) shows that the variance of log-transformed data decreases inversely with peak intensity. This has severe consequences for interpreting the results of PCA. Since PCA orders components by variance, noise in *minor* spectral features may be deemed more important than systematic structure in the data. The 100-pA silver depth profile provides a striking example. Cs^+, which has real depth variation, is discovered in the 47th most significant of the 77 principal components. The PC-47 score clearly tracks the observed depth variation as shown in **Supplementary Figure 16c**, and Cs^+ is the largest contributor to the corresponding loading vector in **Supplementary Figure 16d**. As expected, the leading principal component describes the mean spectrum, however, the instrument-induced small-large cluster anticorrelation is not isolated in any single PC. **Supplementary Figure 16c** also shows the loading for PC 2. The most prominent loading corresponds to $^{107}Ag_9^+$, which accounts for only 0.3% of the total Ag_9^+ intensity in a cluster that itself makes only a minor contribution to the overall silver-cluster distribution. In fact, the three largest loadings describe anticorrelations among the three least intense mass peaks at that peak position. By way of contrast, the least significant PC describes anticorrelation of the two most significant isotopes of Ag_3^+, the most prevalent cluster. Whilst the log transformation does make the mass peaks more comparable, the heteroscedasticity it induces makes the interpretation of PCA highly problematic.

A different transform that has intuitive appeal for count data takes the square root of the data.[27,28] This reflects the fact that the standard deviation of a Poisson random variable is the square root of its mean. The expected mean and variance of a square root transformed data matrix can again be approximated by a Taylor series:

$$E[\sqrt{\mathbf{s}}] = \sqrt{E[\mathbf{s}]} - \frac{1}{8}E[\mathbf{s}]^{-3/2}\mathrm{Var}(\mathbf{s}) = \sqrt{E[\mathbf{s}]} - \frac{1}{8}\left(\frac{A}{\sqrt{E[\mathbf{s}]}} + R_N^2\sqrt{E[\mathbf{s}]}\right)$$
$$\mathrm{Var}(\sqrt{\mathbf{s}}) = \left(\frac{1}{2\sqrt{E[\mathbf{s}]}}\right)^2 \mathrm{Var}(\mathbf{s}) = \frac{1}{4}(A + R_N^2 E[\mathbf{s}]) \quad (44)$$

Supplementary Figure 16e displays the mean of the transformed uncensored subset of the 100-pA dataset. The square root is not as effective as the log transform for equalizing the peak intensities. However, it has the advantage that the transformed data are more nearly

homoscedastic as shown in **Supplementary Figure 16b**, which also includes the model prediction. According to Eq. (44), in the limit that the data is Poisson with no overdispersion, the variance is independent of the mean. PCA on the square-root-transformed data performs well. The three known components are discovered in the 3 leading principal components, and as shown in **Supplementary Figure 16f**, Cs^+ is clearly isolated in PC 3.

Whilst the square-root transform performed nicely with the uncensored silver depth profile data, it has several drawbacks, in general. Recall that root-mean scaling involves scaling each mass spectrum by the inverse square root of the mean spectrum. The number of parameters accounting for the noise is equal to the number of peaks in the spectrum and each parameter is a statistical summary of all spectra in the dataset. Taking the square root of each element in the data matrix is equivalent to scaling each element by its individual inverse square root. The number of noise parameters is greatly inflated to equal the size of the dataset, and each has much greater uncertainty. This makes the method prone to overfitting. A related problem, one we avoided in the example, is the potentially large numbers of zero-intensity elements in the data matrix due to thresholding. Of course, taking the square root of zero is a legitimate operation. However, from the individual scaling point of view, this is equivalent to giving infinite weight to the zeros, which is not the intent. It should also be kept in mind that PCA components derived from transformed data do not reside in “physical” space. To make any absolute comparisons, such as isotope ratios, the components must be back transformed. All these issues can be addressed. For instance, zeros could be replaced by random numbers drawn from the appropriate Rayleigh distribution, or alternative variance-stabilization transforms, such as the Anscombe[29] transform, could be employed. However, a complete discussion is beyond the scope of the paper.

Finally, the original paper[19] describing the scaling method for Poisson data in ToF-SIMS advocated scaling in the spatial domain as well as in the spectral domain. That is, an additional error covariance matrix is defined and is used to scale the rows of the data matrix (depth or image dimension). Experience has shown that this does not have a significant effect unless there are very large region-to-region disparities in total-ion intensity. However, total-ion intensity and its variation are described by Eq. (16). These equations have the same form as the mass spectral mean and variance in Eq. (21). By analogy with root-mean scaling, scaling in the spatial domain can be accomplished by scaling the rows of the data matrix by the inverse square roots of the row means. Additional ways to incorporate spatial scaling in multivariate analysis are outlined elsewhere.[23]

Supplementary Figure 17 presents a summary of several preprocessing methods applied to the 50-pA silver depth profile illustrating their ability to equalize noise. The noise variance of the preprocessed data was estimated by PFA and scaled to have unit mean. All three methods assuming count data (**Supplementary Figure 17a**) perform comparably at moderate to high signal levels. The WSoR scaling method is reasonably successful over the entire intensity range, whereas root-mean scaling has increased, yet uniform, variance at low signal levels. Since PCA on the scaled data is still variance-based, the low intensity signals, which are largely noise, will be relatively upweighted. Interestingly, the ratio of the two plateau regions in the root-mean-scaled curve can be computed from the data model and is approximately the noise threshold divided by the ions-to-signal scale factor *A*. This may provide an avenue for estimating these parameters in more complicated datasets. Within the heuristic methods (**Supplementary Figure 17b**), log transform performs poorly and will not be considered further. Variance scaling makes noise variance in the low-intensity peaks appear uniform but the increasingly reduced variance at high signal levels underweights those peaks with respect to noise. Pareto-scaled data does not produce uniform noise over any part of the intensity range. Rather, the noise variance increases approximately with the square root of the mean over most of the range. Unlike variance and root-mean scaling, Pareto scaling down-weights the low-intensity peaks and increasingly up-weights the high-intensity ones.

To evaluate the impact of overdispersion on the performance of the heuristic scaling methods, we simulated 50-pA dataset with both no overdispersion and with RSD at 5 times the observed value. The simulations with Pareto scaling (**Supplementary Figure 17c)** and variance scaling (**Supplementary Figure 17d)** illustrate the limiting cases that for over-dispersed data, Pareto is unbounded at low signal and Variance at high signal. The noise variance of root-mean-scaled data (**Supplementary Figure 17e)** is bounded and insensitive to the extent of overdispersion. Consequently, root-mean scaling would be a good general-purpose approach with the caveat that some sensitivity is lost due to the relative upweighting of noise.

As stated before, the silver sample has adventitious Cs surface contamination from exposure to a Cs sputter beam not used in this study. This manifests in the depth profile data as a steadily declining Cs^+ signal with sputter dose, which provides a simple data set to test the effect of noise bias on multivariate analysis. The extent of Cs surface concentration varies with the 100-pA data set having the most and the 200-pA data set the least. Without noise bias, we expect the leading principal component to resemble the average mass spectrum. The second and third principal components contain any non-linearity information

relating to the silver peaks and the weak but chemically relevant Cs^+ signal. Briefly, as expected for PCA without scaling the Cs^+ is not discovered until PC 11. Whilst with the

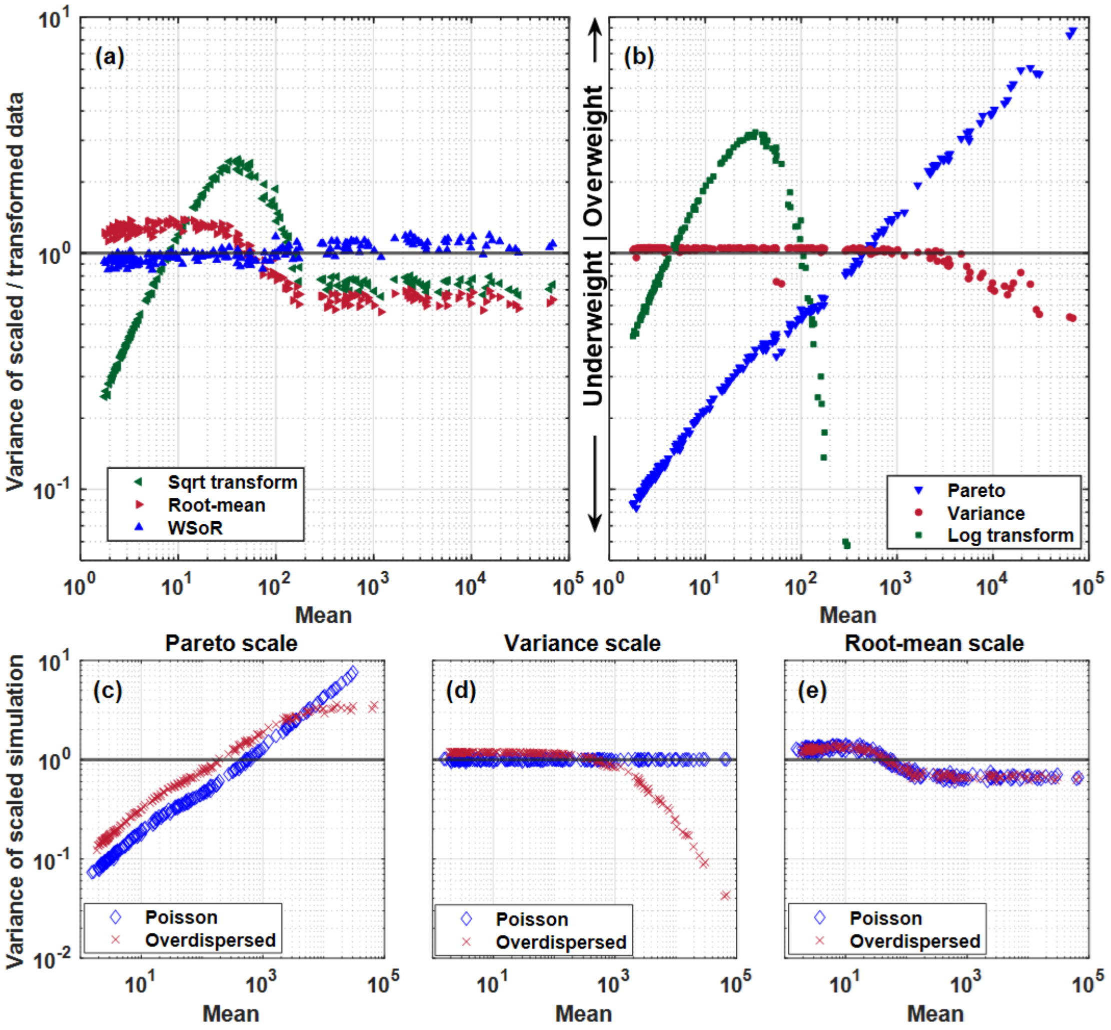

Supplementary Figure 17. Summary of several preprocessing methods applied to the 50-pA silver depth profile illustrating their ability to equalize noise: **a** methods assuming count data. **b** heuristic methods. **c-e** results of simulations to assess sensitivity of scaling methods to overdispersion in the data. Root-mean scaling is a good general-purpose choice as the noise variance is bounded by quantities derived from the data model and it is insensitive to overdispersion.

Supplementary Table 2: Survey of papers (Web of Science) that involve Orbitrap MS and "multivariate analysis" or "PCA" or "Principal Component Analysis".

DOI	Title	Scaling method
10.1016/j.foodchem.2014.08.101	Analysis and characterisation of phytochemicals in mulberry (Morus alba L.) fruits grown in Vojvodina, North Serbia	Limited information
10.1371/journal.pone.0082589	Proteomic Profiling of Exosomes Leads to the Identification of Novel Biomarkers for Prostate Cancer	No scaling information provided
10.1021/ac062455y	Top-down lipidomic screens by multivariate analysis of high-resolution survey mass spectra	No scaling information provided
10.1074/mcp.M111.015362	Super-SILAC Allows Classification of Diffuse Large B-cell Lymphoma Subtypes by Their Protein Expression Profiles	No scaling information provided
10.1007/s00216-011-5540-z	Cord blood metabolomic profiling in intrauterine growth restriction	No scaling information provided
10.1007/s00216-012-5841-x	Analysis of colorectal adenocarcinoma tissue by desorption electrospray ionization mass spectrometric imaging	No scaling information provided
10.1371/journal.pntd.0000904	Metabolomics to Unveil and Understand Phenotypic Diversity between Pathogen Populations	No scaling information provided
10.1016/j.chroma.2012.12.009	Ultra-high pressure liquid chromatography-mass spectrometry for plant metabolomics: A systematic comparison of high-resolution quadrupole-time-of-flight and single stage Orbitrap mass spectrometers	Pareto
10.1021/jf300297y	Structural Annotation and Elucidation of Conjugated Phenolic Compounds in Black, Green, and White Tea Extracts	Log2 transform

10.1002/rcm.4328	Metabolomics profiling of extracellular metabolites in recombinant Chinese Hamster Ovary fed-batch culture	No scaling information provided
10.1007/s00216-011-5540-z	Cord blood metabolomic profiling in intrauterine growth restriction	No scaling information provided
10.1021/ac500476a	A Nano Ultra-Performance Liquid Chromatography-High Resolution Mass Spectrometry Approach for Global Metabolomic Profiling and Case Study on Drug-Resistant Multiple Myeloma	No scaling information provided
10.1021/acs.analchem.5b02254	Untargeted Metabolomics in Doping Control: Detection of New Markers of Testosterone Misuse by Ultrahigh Performance Liquid Chromatography Coupled to High-Resolution Mass Spectrometry	No scaling information provided
10.1371/journal.pone.0103217	Evidence That Multiple Defects in Lipid Regulation Occur before Hyperglycemia during the Prodrome of Type-2 Diabetes	Not clear but maybe log transform
10.1016/j.phytochem.2017.04.005	Chemodiversity of two closely related tetraploid Centaurium species and their hexaploid hybrid: Metabolomic search for high-resolution taxonomic classifiers	Mean centred. Variance scaled

4

Supplementary Note 8: extended experimental details of biological imaging datasets

OrbiSIMS Drosophila CNS dataset: *Drosophila* larvae (*w^{1118} iso31* strain) were raised on a yeast-cornmeal diet (66.3 g/L cornmeal, 58.5 g/L glucose, 23.4 g/L dried inactivated yeast, 7.02 g/L agar, 1.95 g/L Nipagin, and 7.8 mg/L Bavistan) until late L3 stage. A larval central nervous system (CNS) was dissected in phosphate buffered saline (PBS) and fixed in 4% paraformaldehyde in PBS for 30 min at room temperature. After fixation, the sample was washed in PBS and stained with 13.1 µM Toluidine Blue O for 30 min at room temperature, followed by a PBS wash. The CNS was then embedded in 4% carboxymethyl cellulose (sodium salt) in PBS and flash frozen in a bath of dry ice and 2-methylbutane. Larval CNS transverse sections were cut using a Leica CM3050 S Cryostat (Leica Microsystems, Wetzlar, Germany) set to 10 µm thickness, and −20°C chamber and object temperatures. Tissue slices were thaw mounted onto an ITO-coated glass slide (25 mm x 25 mm) with a resistivity of 70-100 Ω/sq (Sigma-Aldrich, #703176), cleaned with sequential washes in 70:30 acetone:water, 2:1 chloroform:methanol and then hexane, and subsequently vacuum packed and stored at -80°C until analysis. Before unsealing the sample, it was allowed to warm to room temperature for 20 mins to reduce condensation on the sample surface. The CNS section was then analysed on an OrbiSIMS (Hybrid SIMS, IONTOF GmbH) instrument. The analysis was performed at ~25°C using a 20 keV Ar_{3500}^{+} quasi-continuous GCIB analysis beam with a spot size of ~3 µm using a sawtooth raster mode, a current of ~12 pA, a duty cycle of 15 % and a cycle length of 200 µs. The total ion dose the image was 8.88 × 10^{14} ions cm^{-2} with 14800 shot per pixel. The image area was 250 µm × 250 µm, with a 5 µm pixel size. The Orbitrap MS was operated in negative-ion polarity with a mass resolution of 240,000 @ 200 *m/z* and an injection time of 2961 ms, with the automatic gain control switched off. The surface potential was 6.1 V. The collisional cooling pressure was set to low with a pressure of 4.3 x 10^{-2} mbar. Mass spectral information was acquired for the range *m/z* 100 – 1,000. The OrbiSIMS instrument was controlled using SurfaceLab, integrating an application programming interface provided by ThermoFisher Scientific. All images and graphs were obtained using centroid data. Prior to PCA, a mask was applied to remove the media/substrate around the cross section, totalling 930 pixels in the dataset. The intensities in the dataset are in the low intensity to medium intensity regime, meaning that there is significant thresholding and relatively higher variance. PCA was calculated using MATLAB functions developed in-house and PFA computed with code available on the Mathworks File Exchange.[12] Pixels with zero intensities were ignored for the log transformation. All results were rotated to the standard PCA basis to enable better comparison.

OrbiSIMS mouse testis dataset: animal studies were performed under a UK Home office approved project license (PAA689E24) and in accordance with institutional welfare guidelines and local ethical committees. All efforts were made to ameliorate any suffering. Male mice (strain FVBN/J, wildtype, 6 – 8 weeks) were housed in a temperature-controlled room at 21°C with a 12-hour light: dark cycle. Water and food were provided *ad libitum.* Mice were killed by cervical dislocation and testes dissected and frozen in liquid nitrogen and stored at -80°C before sectioning. Mouse testis sections were attached to the cryotome chuck using water and blue roll tissue, and cut using a Thermo Scientific NX70 Cryostar (Thermo Scientific, Runcorn, United Kingdom) set to 10 µm thickness, with ~-25°C knife and ~-10°C object temperatures. Tissue slices were thaw mounted onto an ITO-coated glass slide (25 mm x 75 mm) with a resistivity of 70 – 100 Ω/sq (Sigma-Aldrich, #576352), and subsequently vacuum packed and stored at -80°C until analysis. Before unsealing the sample, it was allowed to warm to room temperature for 10 mins to reduce condensation on the sample surface. The testis section was then analysed on an OrbiSIMS (Hybrid SIMS, IONTOF GmbH) instrument. The analysis was performed at ~25°C using a 20 keV Ar_{2500}^+ quasi-continuous GCIB analysis beam with a spot size of ~5 µm using a sawtooth raster mode, a current of ~1.44 pA, a duty cycle of 10 % (lower to previous experiments due to a misalignment) and a cycle length of 200 µs. The total ion dose density of the image was 1.1 × 10^{14} ions cm^{-2} with 2500 shot per pixel. The image area was 200.00 µm × 200.00 µm, with a 2 µm pixel size. The Orbitrap was operated in negative-ion polarity with a mass resolution of 240 000 @ 200 *m/z* and an injection time of 511 ms, with the automatic gain control switched off. The surface potential was -30V. The collisional cooling was set to low with a He pressure of 4.2 x 10^{-2} mbar. Mass spectral information was acquired for the range *m/z* 100 – 1500 u. The OrbiSIMS instrument was controlled using SurfaceLab, integrating an application programming interface provided by ThermoFisher Scientific. All images and graphs were obtained using centroid data.

Supplementary Note 9: Additional figures

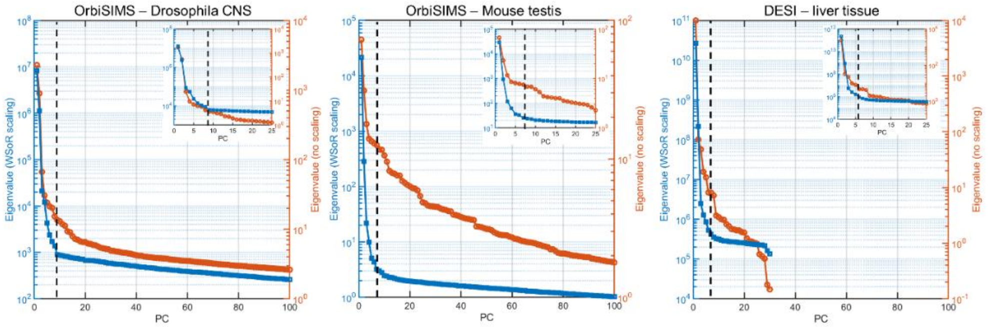

Supplementary Figure 18. PCA eigenvalue plots for the 3 biological datasets with no scaling (red) and WSoR scaling (blue).

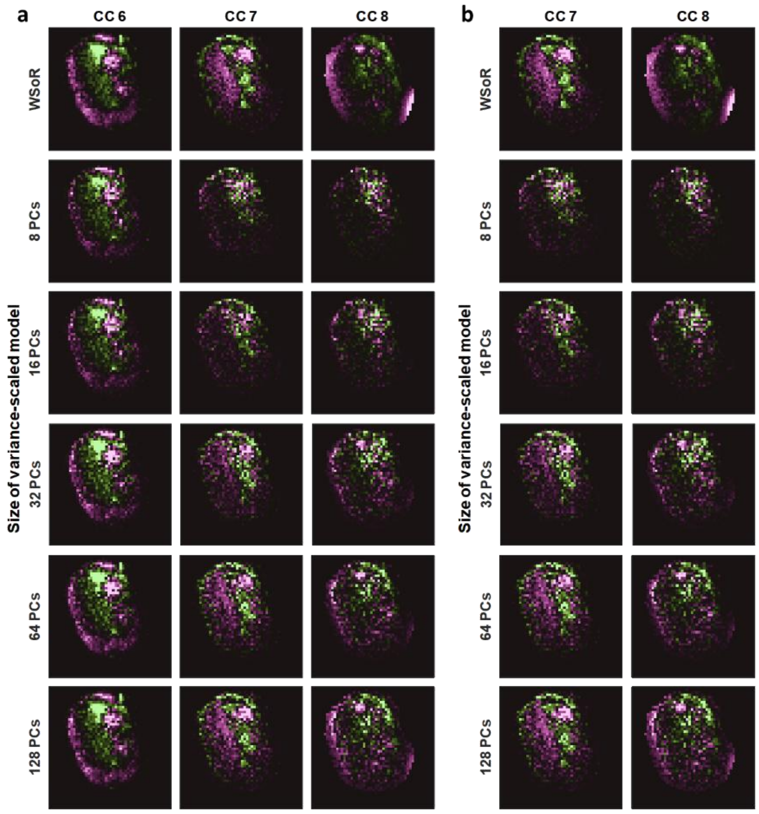

Supplementary Figure 19. CNS Dataset**: P**rincipal scores as a function of model size to show that several tens of components will be required before the Variance-scaled model captures the information in WSoR PCs 7 (**a**) and 8 (**b**) in a recognizable form.

Supplementary References

1. Gudbjartsson, H. & Patz, S. The rician distribution of noisy mri data. *Magn. Reson. Med.* **34**, (1995).
2. Rice, S. O. Mathematical Analysis of Random Noise. *Bell Syst. Tech. J.* **24**, (1945).
3. Aja-Fernández, S. & Vegas-Sánchez-Ferrero, G. *Statistical Analysis of Noise in MRI*. (Springer International Publishing, 2016). doi:10.1007/978-3-319-39934-8.
4. Zhurov, K. O., Kozhinov, A. N., Fornelli, L. & Tsybin, Y. O. Distinguishing analyte from noise components in mass spectra of complex samples: Where to cut the noise? *Anal. Chem.* **86**, 3308–3316 (2014).
5. Pishro-Nik, H. Introduction to Probability, Statistics, and Random Processes. *Kappa Res. LLC* (2014).
6. Ross, S. *Introduction to probability models*. (2019). doi:10.1016/C20170013241.
7. Wiuf, C. & Stumpf, M. P. H. Binomial subsampling. *Proc. R. Soc. A Math. Phys. Eng. Sci.* **462**, (2006).
8. Ross, S. M. *A first course in probability*. (Pearson, 2008).
9. Bishop, C. M. *Pattern Recognition and Machine Learning*. (Springer, 2006).
10. Roweis, S. EM Algorithms for PCA and SPCA. *Computing* **10**, 626–632 (1997).
11. Tipping, M. E. & Bishop, C. M. Probabilistic principal component analysis. *J. R. Stat. Soc. Ser. B Stat. Methodol.* **61**, (1999).
12. Mo Chen (2023). Probabilistic PCA and Factor Analysis (https://www.mathworks.com/matlabcentral/fileexchange/55883-probabilistic-pca-and-factor-analysis), MATLAB Central File Exchange. Retrieved April 13, 2023.
13. Halko, N., Martinsson, P. G. & Tropp, J. A. Finding Structure with Randomness : Probabilistic Algorithms for Matrix Decompositions **53**, 217–288 (2011).
14. Leger, M. N., Vega-Montoto, L. & Wentzell, P. D. Methods for systematic investigation of measurement error covariance matrices. *Chemom. Intell. Lab. Syst.* **77**, (2005).
15. Bowsher, C. G. & Swain, P. S. Identifying sources of variation and the flow of information in biochemical networks. *Proc. Natl. Acad. Sci. U. S. A.* **109**, (2012).
16. Sibuya, M., Yoshimura, I. & Shimizu, R. Negative multinomial distribution. *Ann. Inst. Stat. Math.* **16**, (1964).
17. Zhou, H. & Lange, K. MM algorithms for some discrete multivariate distributions. *J. Comput. Graph. Stat.* **19**, (2010).
18. R. Malek, O. Lange, Method of Processing and Storing Mass Spectrometry Data, Patent: US 7,657,387 B2, February 2, 2010, Assignee: Thermo Finnigan LLC, USA.
19. Keenan, M. R. & Kotula, P. G. Accounting for Poisson noise in the multivariate analysis of ToF-SIMS spectrum images. *Surf. Interface Anal.* **36**, 203–212 (2004).
20. Keenan, M. R. & Smentkowski, V. S. The statistics of ToF-SIMS data revisited and introduction of the empirical Poisson correction. *Surf. Interface Anal.* **48**, 218–225 (2016).

21. Switzer, P. & Green, A. A. *Min/max autocorrelation factors for multivariate spatial imagery*. (1984).
22. Tyler, B. J., Rayal, G. & Castner, D. G. Multivariate analysis strategies for processing ToF-SIMS images of biomaterials. *Biomaterials* **28**, 2412–2423 (2007).
23. Keenan, M. R., Windig, W. & Arlinghaus, H. Framework for alternating-least-squares-based multivariate curve resolution with application to time-of-flight secondary ion mass spectrometry imaging. *J. Vac. Sci. Technol. A Vacuum, Surfaces, Film.* **33**, 05E123 (2015).
24. Withers, C. S. & Nadarajah, S. The spectral decomposition and inverse of multinomial and negative multinomial covariances. *Brazilian J. Probab. Stat.* **28**, (2014).
25. van den Berg, R. A., Hoefsloot, H. C. J., Westerhuis, J. A., Smilde, A. K. & van der Werf, M. J. Centering, scaling, and transformations: Improving the biological information content of metabolomics data. *BMC Genomics* **7**, (2006).
26. Eriksson, L., Byrne, T., Johansson, E., Trygg, J. & Vikström, C. Multi- and Megavariate Data Analysis Basic Principles and Applications. *Technometrics* (2013) doi:10.1198/tech.2003.s162.
27. Purohit, P. V. & Rocke, D. M. Discriminant models for high-throughput proteomics mass spectrometer data. in *Proteomics* vol. 3 (2003).
28. Tang, Y. & Harrington, P. B. Noninteger Root Transformations for Preprocessing Nanoelectrospray Ionization High-Resolution Mass Spectra for the Classification of Cannabis. *Anal. Chem.* **91**, (2019).
30. Björck, Åke & Golub, G. H. Numerical methods for computing angles between linear subspaces. *Math. Comput.* **27**, (1973).
31. Keenan, M. R. Exploiting spatial-domain simplicity in spectral image analysis. *Surf. Interface Anal.* **41**, (2009).
