# Supplementary material for: Orbitrap noise structure and method for noise unbiased multivariate analysis
Source: Nat Commun. 2025 Jul 10;16:6398. doi: 10.1038/s41467-025-61542-2 (PMC12246260; doi:10.1038/s41467-025-61542-2)
Supplement: Supplementary file 2 — Reporting Summary [file 41467_2025_61542_MOESM2_ESM.pdf]

nature portfolio

Corresponding author(s): Ian S. Gilmore

Last updated by author(s): Feb 15, 2024

Reporting Summary

Nature Portfolio wishes to improve the reproducibility of the work that we publish. This form provides structure for consistency and transparency in reporting. For further information on Nature Portfolio policies, see our Editorial Policies and the Editorial Policy Checklist.

Statistics

For all statistical analyses, confirm that the following items are present in the figure legend, table legend, main text, or Methods section.

n/a	Confirmed	
☐	☒	The exact sample size (*n*) for each experimental group/condition, given as a discrete number and unit of measurement
☒	☐	A statement on whether measurements were taken from distinct samples or whether the same sample was measured repeatedly
☒	☐	The statistical test(s) used AND whether they are one- or two-sided<br>*Only common tests should be described solely by name; describe more complex techniques in the Methods section.*
☒	☐	A description of all covariates tested
☒	☐	A description of any assumptions or corrections, such as tests of normality and adjustment for multiple comparisons
☐	☒	A full description of the statistical parameters including central tendency (e.g. means) or other basic estimates (e.g. regression coefficient) AND variation (e.g. standard deviation) or associated estimates of uncertainty (e.g. confidence intervals)
☒	☐	For null hypothesis testing, the test statistic (e.g. *F*, *t*, *r*) with confidence intervals, effect sizes, degrees of freedom and *P* value noted<br>*Give P values as exact values whenever suitable.*
☒	☐	For Bayesian analysis, information on the choice of priors and Markov chain Monte Carlo settings
☒	☐	For hierarchical and complex designs, identification of the appropriate level for tests and full reporting of outcomes
☒	☐	Estimates of effect sizes (e.g. Cohen's *d*, Pearson's *r*), indicating how they were calculated

Our web collection on statistics for biologists contains articles on many of the points above.

Software and code

Policy information about availability of computer code

Data collection	Data was collected using instrument manufacturer's software Surface Lab 7 (IONTOF GmbH) and Tune (Thermo-Fisher Scientific)
Data analysis	Data analysis was done using instrument manufacturer's software Surface Lab 7 (IONTOF GmbH) and MATLAB R2023b (Mathworks)

For manuscripts utilizing custom algorithms or software that are central to the research but not yet described in published literature, software must be made available to editors and reviewers. We strongly encourage code deposition in a community repository (e.g. GitHub). See the Nature Portfolio guidelines for submitting code & software for further information.

Data

Policy information about availability of data

All manuscripts must include a data availability statement. This statement should provide the following information, where applicable:

- Accession codes, unique identifiers, or web links for publicly available datasets
- A description of any restrictions on data availability
- For clinical datasets or third party data, please ensure that the statement adheres to our policy

All OrbiSIMS data reported in this manuscript have been deposited as imzML files to figshare (https://doi.org/10.6084/m9.figshare.28684622). imzML files can be processed in MATLAB using the imzML parser of SpectralAnalysis (https://github.com/AlanRace/SpectralAnalysis).

Research involving human participants, their data, or biological material

Policy information about studies with human participants or human data. See also policy information about sex, gender (identity/presentation), and sexual orientation and race, ethnicity and racism.

Reporting on sex and gender	N/A
Reporting on race, ethnicity, or other socially relevant groupings	N/A
Population characteristics	N/A
Recruitment	N/A
Ethics oversight	N/A

Note that full information on the approval of the study protocol must also be provided in the manuscript.

Field-specific reporting

Please select the one below that is the best fit for your research. If you are not sure, read the appropriate sections before making your selection.

- [x] Life sciences
- [ ] Behavioural & social sciences
- [ ] Ecological, evolutionary & environmental sciences

For a reference copy of the document with all sections, see nature.com/documents/nr-reporting-summary-flat.pdf

Life sciences study design

All studies must disclose on these points even when the disclosure is negative.

Sample size	2 biological image data sets (each containing 2,500 and 10,000 pixels) (drosophila and mouse) and at least 15 silver data sets, each containing around 1000 measurements.
Data exclusions	Please refer to the manuscript. Exclusions for statistical fits are clearly stated
Replication	We tested the method using 2 biological image data sets (each containing 2,500 and 10,000 pixels) (drosophila and mouse) and have also tested the method on spatially patterned organic molecules. The method could be replicated. We have tested the method using at least 15 silver data sets, each containing around 1000 measurements.
Randomization	N/A
Blinding	N/A

Reporting for specific materials, systems and methods

We require information from authors about some types of materials, experimental systems and methods used in many studies. Here, indicate whether each material, system or method listed is relevant to your study. If you are not sure if a list item applies to your research, read the appropriate section before selecting a response.

Materials & experimental systems

n/a	Involved in the study	
[x]	[ ]	Antibodies
[x]	[ ]	Eukaryotic cell lines
[x]	[ ]	Palaeontology and archaeology
[ ]	[x]	Animals and other organisms
[x]	[ ]	Clinical data
[x]	[ ]	Dual use research of concern
[x]	[ ]	Plants

Methods

n/a	Involved in the study	
[x]	[ ]	ChIP-seq
[x]	[ ]	Flow cytometry
[x]	[ ]	MRI-based neuroimaging

Animals and other research organisms

Policy information about studies involving animals; ARRIVE guidelines recommended for reporting animal research, and Sex and Gender in Research

Laboratory animals	Drosophila larvae (w1118 iso31 strain) were raised on a yeast-cornmeal diet (66.3 g/L cornmeal, 58.5 g/L glucose, 23.4 g/L dried inactivated yeast, 7.02 g/L agar, 1.95 g/L Nipagin, and 7.8 mg/L Bavistan) until late L3 stage.
Wild animals	Animal studies were performed under a UK Home office approved project license (PAA689E24) and in accordance with institutional welfare guidelines and local ethical committees. All efforts were made to ameliorate any suffering. Mice were killed by cervical dislocation and testes dissected and frozen in liquid nitrogen and stored at -80°C before sectioning
Reporting on sex	Sex is reported but no sex was considered for hypotheses and conclusions
Field-collected samples	Male mice (strain FVBN/J, wildtype, 6 – 8 weeks) were housed in a temperature-controlled room at 21°C with a 12-hour light: dark cycle. Water and food were provided ad libitum.
Ethics oversight	Animal studies were performed under a UK Home office approved project license (PAA689E24)

Note that full information on the approval of the study protocol must also be provided in the manuscript.

Plants

Seed stocks	N/A
Novel plant genotypes	N/A
Authentication	N/A
